# Supplementary figures and images for: A toolbox of engineered mosquito lines to study salivary gland biology and malaria transmission
Source: PLoS Pathog. 2022 Oct 12;18(10):e1010881. doi: 10.1371/journal.ppat.1010881 (PMC9555648; doi:10.1371/journal.ppat.1010881)

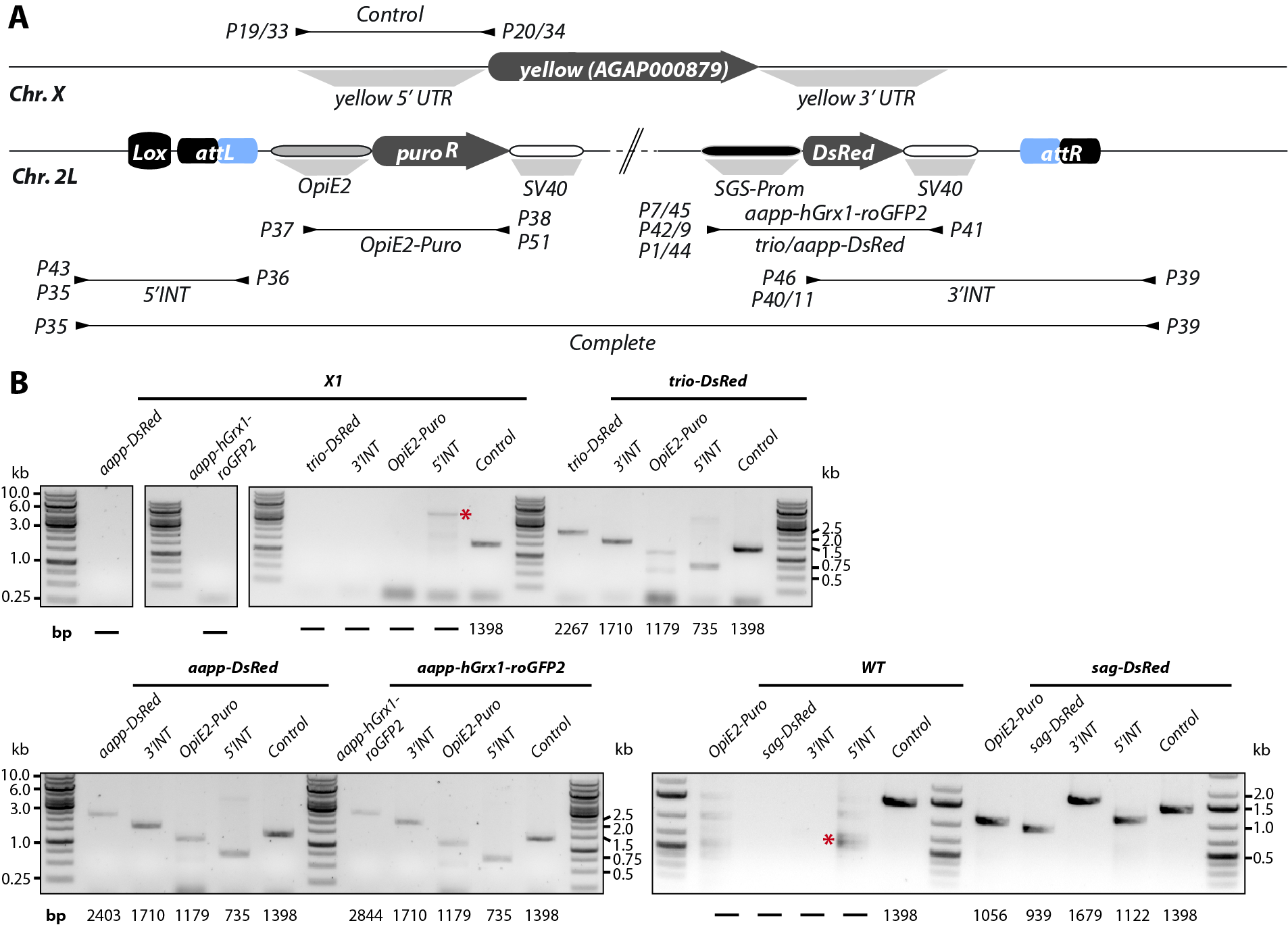

Supplement: S1 Fig — A) Schematic representation of salivary gland reporter transgenesis cassettes inserted on chromosome 2L. Fusion of attB and attP sites generate attL and attR sites after integration. The Lox site upstream of the transgene is a remnant of the fluorescence cassette initially required to select the docking line X1. Note that the illustration is not drawn to scale. B) Genotyping of generated transgenic mosquito colonies in comparison to wild type (WT: G3) or the parental line X1. Five different analytical PCRs were performed. The 5’INT and 3’INT PCRs amplify the integration borders upstream and downstream of the transgene, respectively. The presence of the puromycin resistance cassette was tested (OpiE2-Puro PCR). To ensure integration of the relevant fluorescence cassette, the PCRs aapp-DsRed, aapp-hGrx1-roGFP2, trio-DsRed and sag-DsRed were performed. In addition, a PCR control amplifying the 5’UTR of the yellow gene on chromosome X was included. Genotyping primers and amplified sequences are indicated with arrowheads and thin black lines, respectively, in scheme (A). Expected amplicon sizes are indicated under the gel images, (-) indicates that no amplification is expected. Red asterisks mark unspecific amplicons. (TIF) [file ppat.1010881.s001.tif]

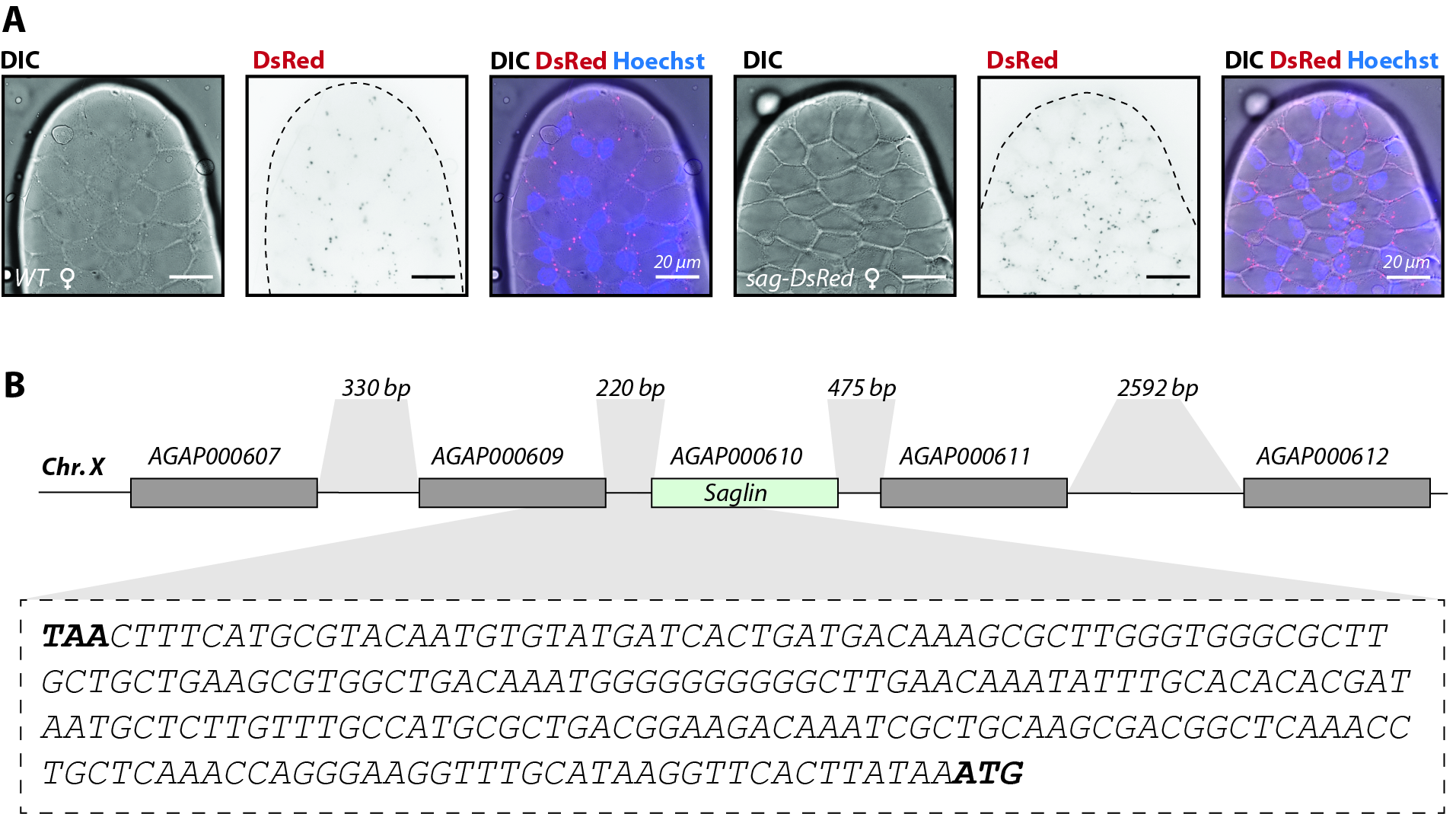

Supplement: S2 Fig — A) Images of the apex of the salivary gland median lobe from a sag-DsRed and a wild type female (Ngousso). Dissected glands were stained with Hoechst 33342 to detect the nuclei of acinar cells. The dotted black line in DsRed images indicates the outline of the salivary gland lobe observed in DIC. Scale bar: 20 μm. B) Genomic context of saglin (AGAP000610) with neighboring genes. The length of intergenic sequences is indicated above the scheme and the sequence tested for promoter activity located between AGAP000609 and saglin is shown. The stop codon TAA of AGAP000609 and the start codon of saglin are highlighted in black. The length of the cloned sequenced intergenic sequence was 220 bp (without stop codon of AGAP000609 and start codon of saglin). Note that the illustration is not drawn to scale. (TIF) [file ppat.1010881.s002.tif]

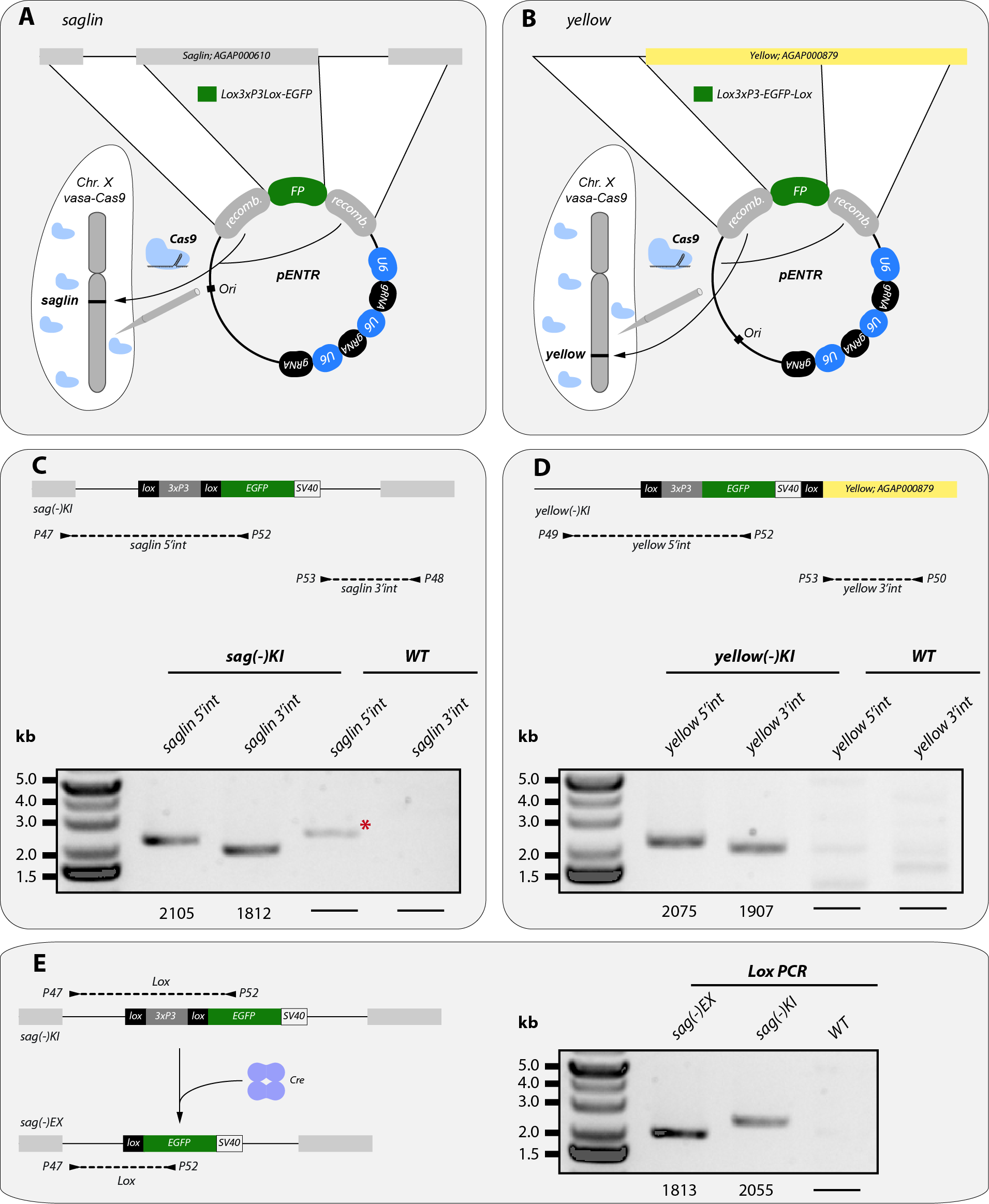

Supplement: S3 Fig — Sag(-)KI (A) and yellow(-)KI (B) mosquitoes were generated by injecting embryos expressing Cas9 (vasa-Cas9) with plasmids carrying repair templates that contain a Lox3xP3Lox-EGFP or a Lox3xP3-EGFP-Lox cassette flanked with upstream and downstream sequences of the respective targeted loci, in combination with three guide RNAs specific for the saglin (AGAP000610) or yellow gene (AGAP000879). Genotyping of the 5’ and 3’ integration borders revealed successful integration of the transgenes into saglin (C) and yellow (D). The red asterisk marks an unspecific PCR product observed in the wild type control (WT: Ngousso). E) To investigate the native expression of the saglin promoter, the 3xP3 promoter initially used to select transgenic sag(-)KI mosquito larvae was removed by Cre-mediated excision. The loss of the 3xP3Lox sequence in sag(-)EX mosquitoes was confirmed by PCR. Expected amplicon sizes are indicated under the gel images, (-) indicates that no amplification is expected (C, D, E). (TIF) [file ppat.1010881.s003.tif]

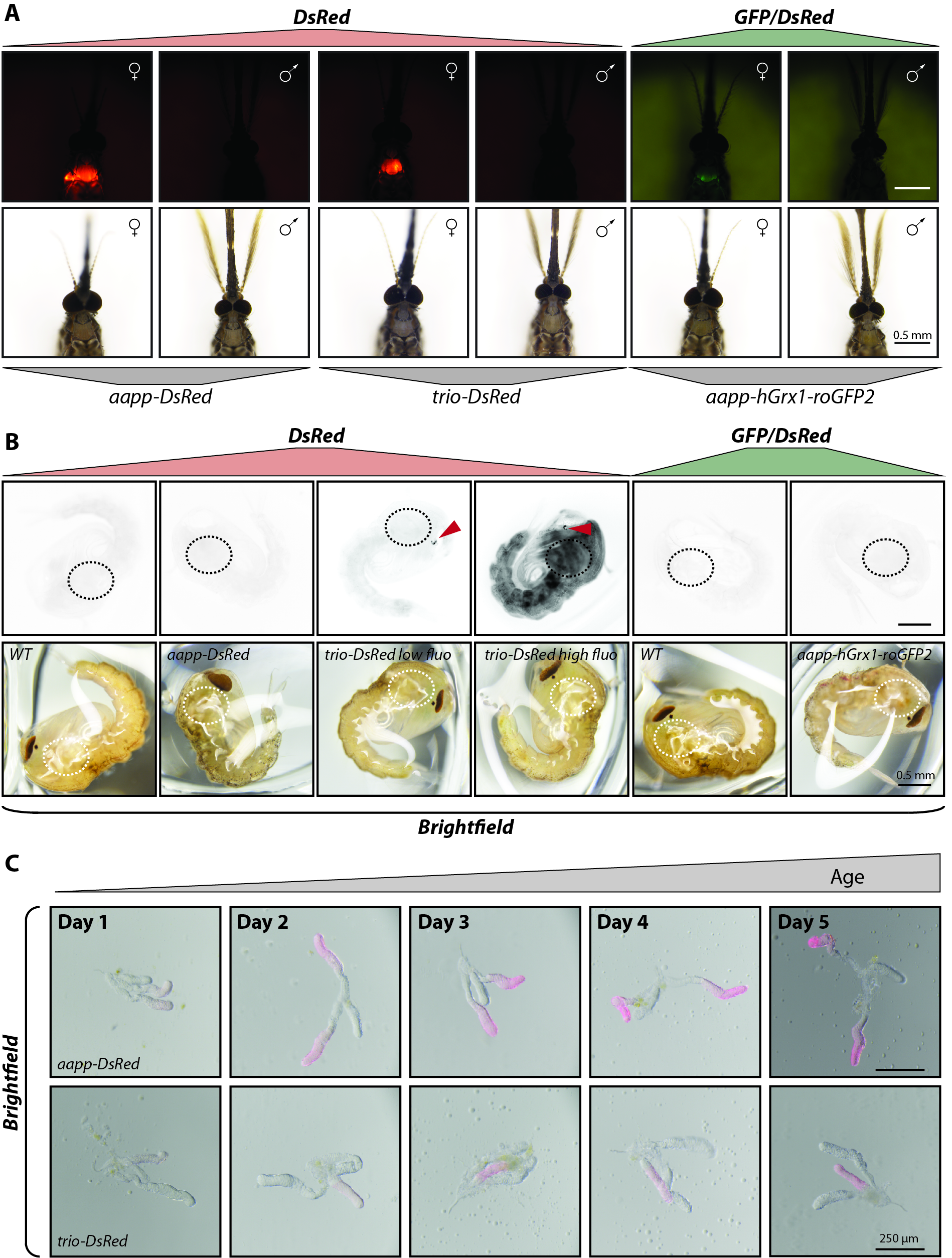

Supplement: S4 Fig — A) Fluorescence and brightfield images of female / male pairs of trio-DsRed, aapp-DsRed and aapp-hGrx1-roGFP2 mosquitoes. Note that all depicted mosquitoes were bred in synchrony and were seven day old (+/- 1 day). Scale bar: 0.5 mm. B) Fluorescence and brightfield images of aapp-DsRed, trio-DsRed and aapp-hGrx1-roGFP2 pupae in comparison to wild type (G3). Dotted circles indicate the putative position of the salivary glands. Trio-DsRed pupae with low and high DsRed body fluorescence are shown for comparison. The red arrows indicate DsRed positive ocelli observed in most trio-DsRed pupae. Note that the fluorescence and brightfield images do not fully overlap as images pupae were able to move between the two pictures. Scale bar: 0.5 mm. C) Brightfield images of aapp-DsRed and trio-DsRed female salivary glands from day 1 to day 5 after hatching (a single image per day and per line). All images were acquired using the same settings. Scale bar: 250 μm. (TIF) [file ppat.1010881.s004.tif]

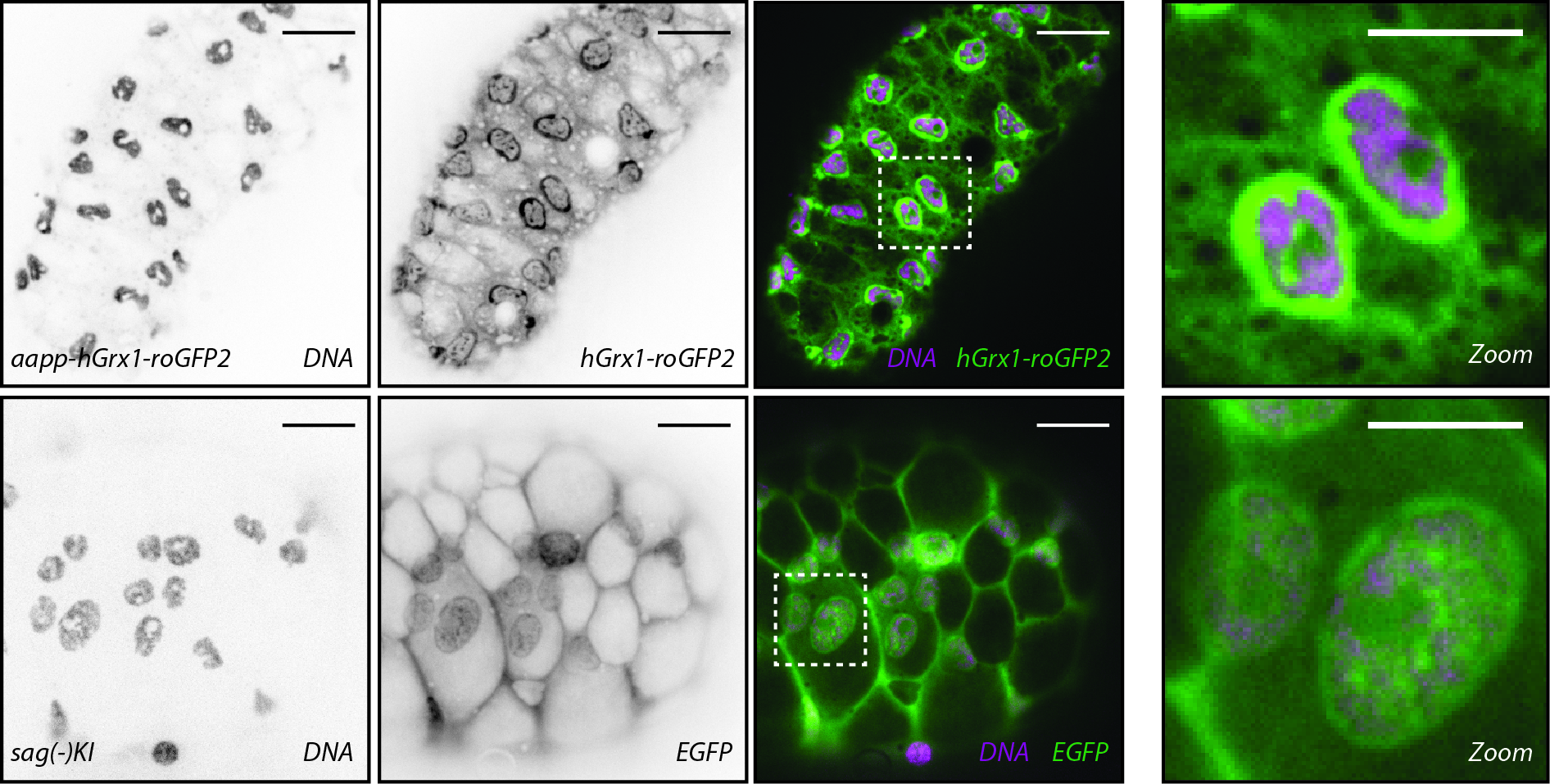

Supplement: S5 Fig — Dissected salivary glands from aapp-hGrx1-roGFP2 and sag(-)KI were stained with Hoechst 33342 (DNA) and imaged by confocal microscopy. The sag(-)KI line was chosen instead of the sag(-)EX line as it displays the same EGFP pattern but with stronger signal. Distal-lateral (top) and median (bottom) lobes are shown for aapp-hGrx1-roGFP2 and sag(-)KI, respectively. Left to right: DNA and EGFP signals in black on white, combination of both signals in colors, and zooms of cells framed in the previous images. Scale bars: 20 μm except for zooms (10 μm). (TIF) [file ppat.1010881.s005.tif]

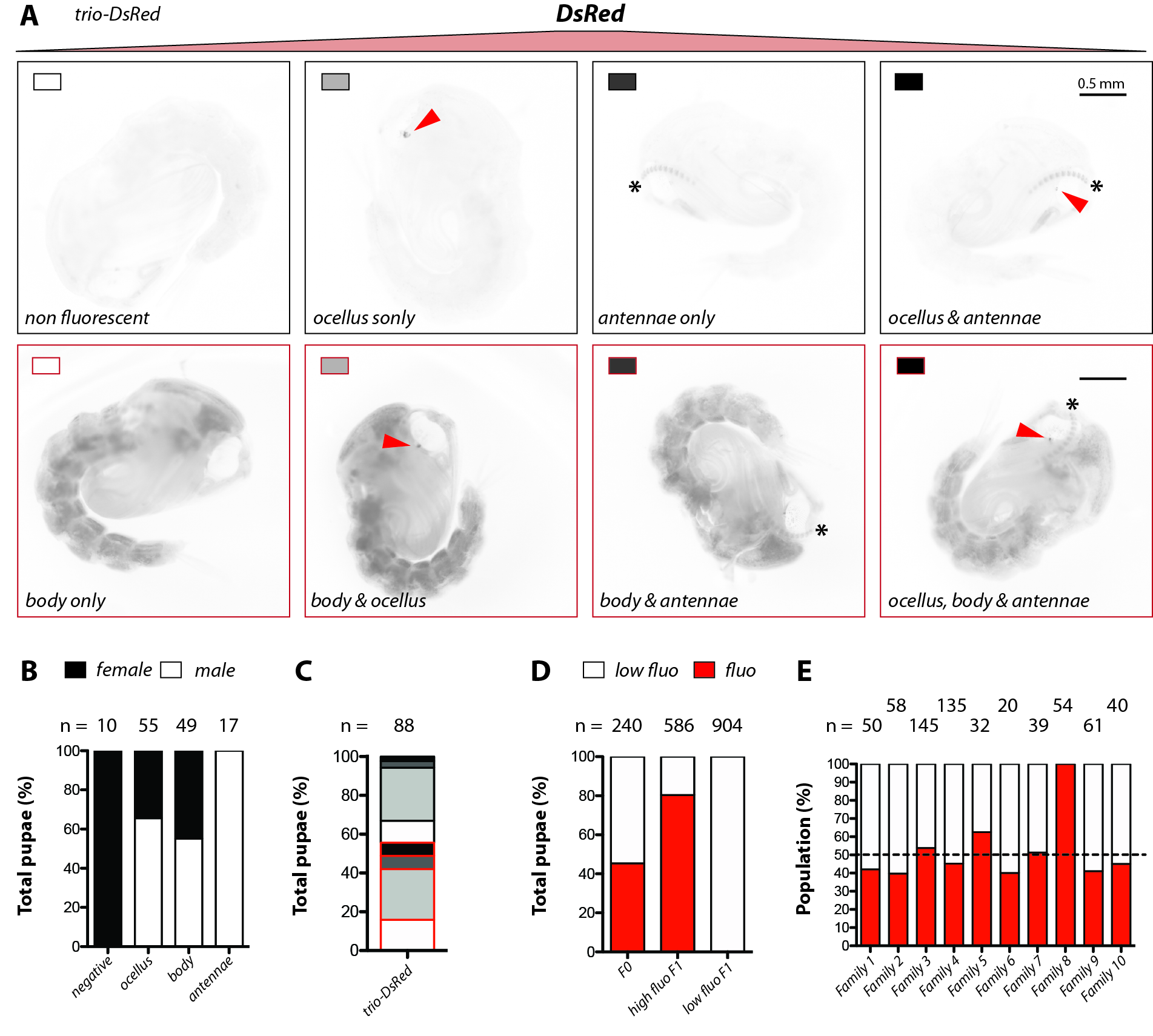

Supplement: S6 Fig — A) Representative images of DsRed expression patterns in trio-DsRed pupae. A subpopulation of pupae displayed high body DsRed fluorescence (images in red frames) while remaining pupae showed no DsRed body fluorescence (black frames). Some pupae displayed fluorescent ocelli and/or fluorescent antennae. The presence of DsRed expression in body, ocelli and antennae occurred in different combinations that are represented by rectangles with different shades and frames. Scale bar: 0.5 mm B) Fluorescence patterns in pupae in relation to their sex. Number of pupae indicated above columns. Data pooled from four generations (≥20 pupae per generation). C) Proportion of each fluorescence pattern in relation to the whole population. Shading and framing of the different patterns as in (A). D) Proportion of pupae with high and low body fluorescence in the trio-DsRed colony (F0) and after intercrossing individuals displaying high or low pupal body fluorescence (F1). The number of analyzed individuals is given above each column. E) Females hatched from pupae with high body fluorescence analyzed in (D, high fluo F1) were kept separately and crossed to wild type males (Ngousso). The progeny of single females was evaluated at the pupal stage for body fluorescence (same color code as D, dashed line indicates 50% of the population). (TIF) [file ppat.1010881.s006.tif]

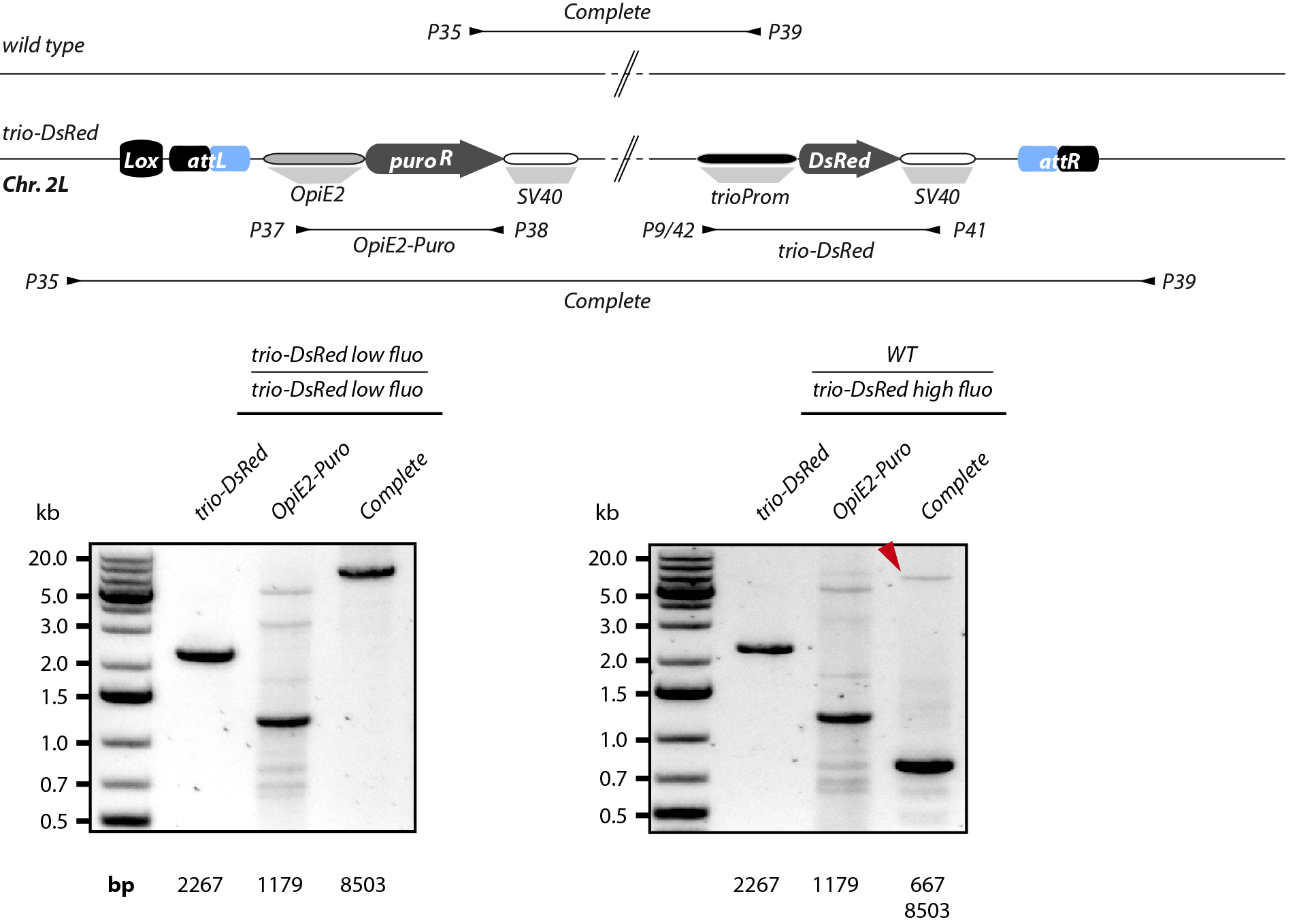

Supplement: S7 Fig — Genotyping of a female mosquito homozygous for the trio-DsRed low fluorescence transgene (trio-DsRed low fluo) taken from the colony generated by the intercross of pupae displaying no body fluorescence (see Fig 4 –Supplement 1D) and of a female mosquito heterozygous for the trio-DsRed high fluorescence transgene (trio-DsRed high fluo) and the wild type allele (WT) obtained from family 1 (Fig 4 –Supplement 1E). An illustration of the modified X1 locus containing the trio-DsRed transgene in comparison to the unmodified wild type allele is shown on top. Genotyping primers and expected products are shown as black arrows and lines, respectively. The length of the expected PCR products is indicated below the gel images. Note that for the heterozygous female carrying the trio-DsRed high fluorescence transgene, the smaller PCR fragment amplifying the unmodified locus dominates while the long PCR fragment representing the transgene containing allele is visible as a faint band at the upper edge (marked by a red arrow). The OpiE2-Puro PCR to verify the presence of the transgene gave several unspecific bands. PCRs were performed on genomic DNA obtained from single mosquitoes. (TIF) [file ppat.1010881.s007.tif]

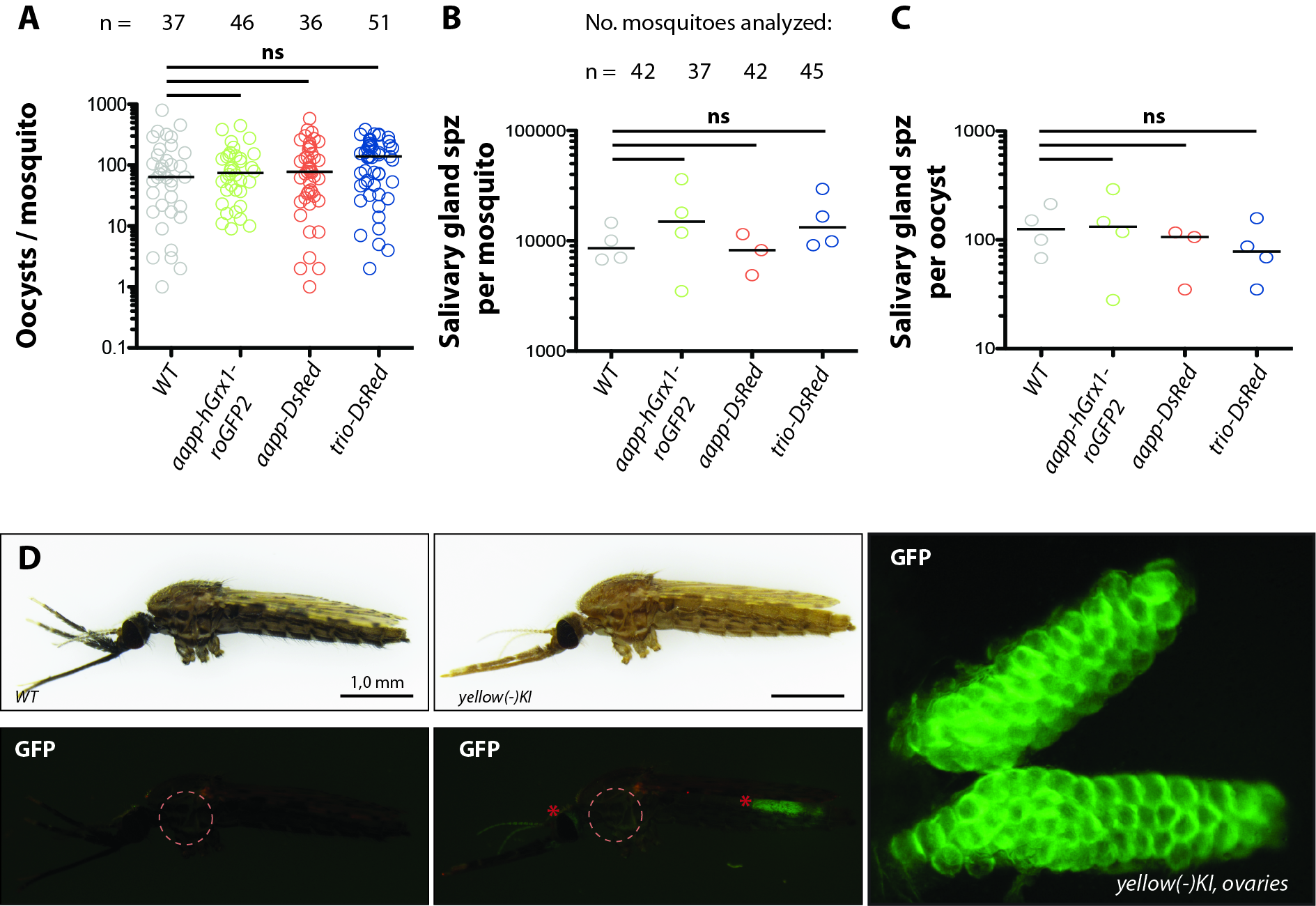

Supplement: S8 Fig — Oocyst (A) and salivary gland sporozoite (spz) (B) counts in infected aapp-DsRed, aapp-hGrx1-roGFP2 and trio-DsRed females in comparison to wild type (G3) females. Data pooled from 3–4 experiments generated with three different mosquito generations. The total number of dissected mosquitoes is given above each genotype. Data points represent parasites counted per midgut (A) and mean number of sporozoites per mosquito in independent experiments (B). C) Number of salivary gland sporozoites (spz) per oocyst for all three reporter lines in comparison to wild type (WT: G3). The invasion rate was calculated using the data shown in (A) and (B). Each dot represents the mean of an independent experiment, the median is indicated by a bar. All data were tested for significance using a Kruskal-Wallis test. ns: not significant (p>0.05). D) GFP background fluorescence of yellow(-)KI in comparison to a wild-type (Ngousso) female. Combinatorial transcriptional activity guided by the 3xP3 and the endogenous yellow promoter drive EGFP expression in the eye and the ovaries of yellow(-)KI females (indicated by red asterisks). No EGFP-like fluorescence was observed in the throat region where the salivary glands localize (indicated by dashed red circle). Scale bar: 1 mm. The image on the right shows EGFP fluorescence from a dissected ovary of a yellow(-)KI female at higher magnification. (TIF) [file ppat.1010881.s008.tif]

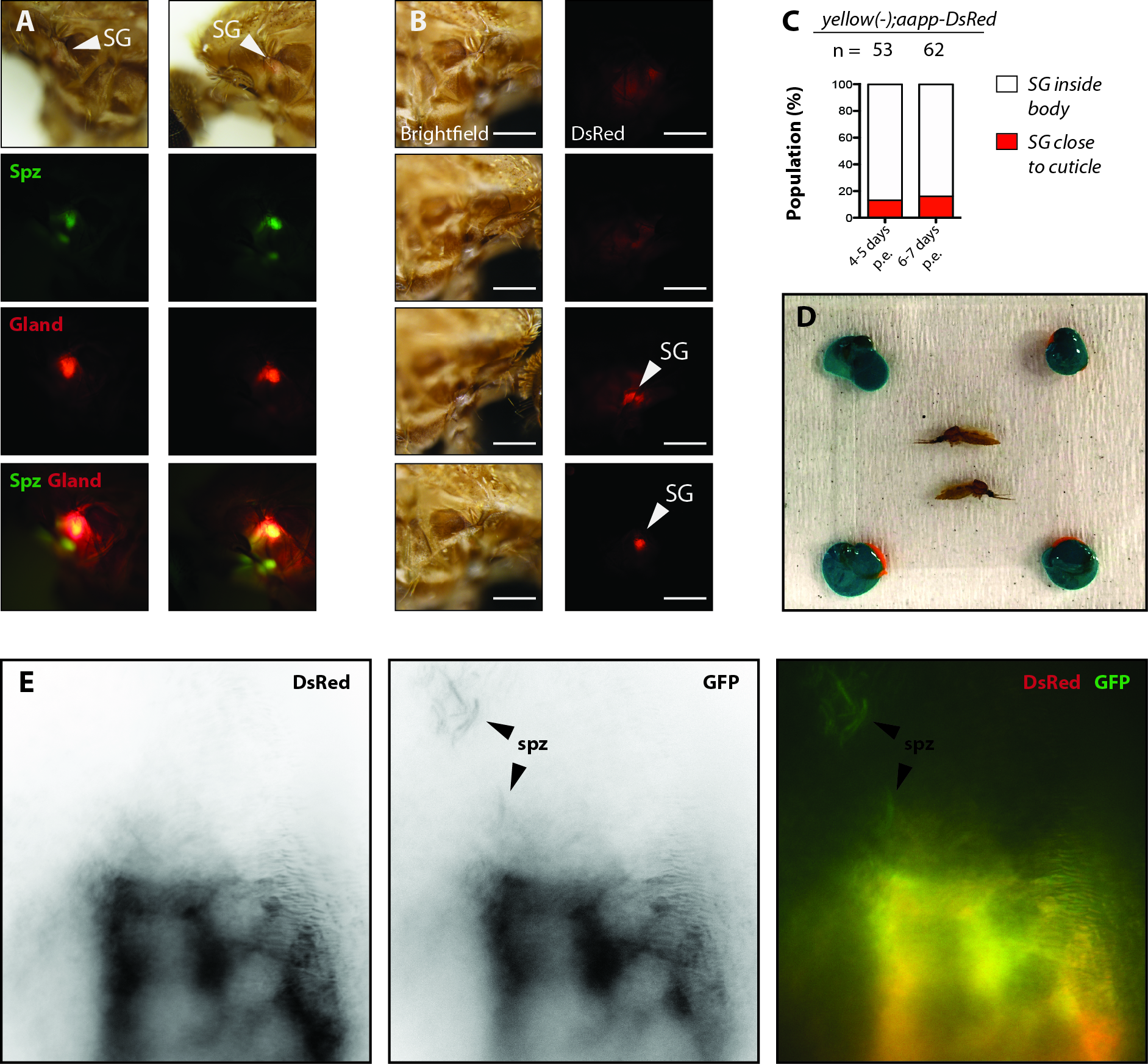

Supplement: S9 Fig — A) Images of two infected mosquitoes with sporozoites (Spz, GFP channel) inside or close to DsRed-expressing salivary glands (SG, red channel). The bottom image was acquired with a filter set visualizing signals of DsRed and GFP. Salivary glands in both mosquitoes localised close to the cuticle. Their position is even visible in brightfield as a light red dot. B) The position of salivary glands varies between mosquitoes, either deep (rows 1 & 2) or close to the cuticle (rows 3 & 4). Brightfield (left) and DsRed (right) images of the same field of view. Images were acquired with the same settings. Scale bar: 250 μm. C) Quantification of salivary gland positioning according to the fluorescence pattern shown in (B). Two mosquito batches were evaluated per time point post emergence (p.e.). The number of evaluated mosquitoes is indicated above each column. D) Sample preparation before imaging. Legless mosquitoes were glued on a microscopy slide and covered with a cover slip underlaid with orange plasticine to avoid squeezing. Corner areas were subsequently coated with teal-colored nail polish to prevent the cover slip from moving. E) DsRed, GFP and merging the two signals of the image shown in Fig 8A. Note that a significant portion of the DsRed signal is also visible in the GFP channel, whereas the sporozoites are exclusively visible in the GFP channel, potentially indicating spillover of the red signal due to the high DsRed concentration. (TIF) [file ppat.1010881.s009.tif]
