# Supplementary material for: A toolbox of engineered mosquito lines to study salivary gland biology and malaria transmission
Source: PLoS Pathog. 2022 Oct 12;18(10):e1010881. doi: 10.1371/journal.ppat.1010881 (PMC9555648; doi:10.1371/journal.ppat.1010881)
Supplement: S1 Appendix — Nucleotides highlighted in red are part of the primer sequence used to amplify the promoter sequence. Sequences with a grey background indicate the 5’UTRs of the trio and aapp transcript, respectively. (DOCX) [file ppat.1010881.s012.docx]

***aapp* promoter sequence (AGAP009974, 1673 bp):**

CTTTTCTTTTACCCTTTGTAACACGCTAATAACGAAAGACCTCCTGCGTGTTAGTACGTGGTGAGGATGATGCGTTTTAGCCTAGCTAGTATTCAAAAAACCTATTAATGCAACAATTACAGTGTGAGGTTACAAAATGGAATTAATTAAACACACTGCTCAACACAGCCATCGTGCTGCATCGTGCTTTGTTGAGGCGCAACAGTTAAATTGTAAGTGCAACCACCGGTATAGAGAGCTGCTTTTGGCAAATGATATGGAATTGTGCTAAATTATGTAATGTTTTTTTTAACTAACTCGTTTACCCGTTAATGGTGGAATGTTTGCAGGTGCGTAGATTAATTTTTTTTAAATGTGCAAATGGAGAAAACAGGATAACGCAAAAGTATTTCCTTCGCTGTAGTAATAGGCTATTATTTATGTACGTCTTTATGTGACCCCGCTAGTGTTGGCTGTATTTTATATCTGTAGGTAAATGACGCGCTATGTATTGTAATAAAATTGGACAAACATTTGCATGTACGATCTGTTAGCTTAGAGAATAAGTATTTTTCTTTTTCGCTACATTTCTCTAAATTCGACCGACGATGACTGTTTTAGGTGTTTTTTCGTAACTTGATCAAGGTAACACCTTTTCTTGGCGTAACGTCCTACGCGGACATGCCGGCCGGCCTTATTCAGGCTTTCGAGACTTAATTCATTACCACGTAGCCGGATAAGTCCAATCCTTGCACAACGGGGGAACGGGTGTCCATTTCAGGCTTGGGCCCATGACGGGCATGTTATTGAGTCGTTCGAGTTGACGACTGTACCATGGGACCGCCCCATTTTTGGCAGTCCATTGATCGTTACGTCGTTTTACGTCTACTGAAGGATGTCCGTTATCAGGTCAAGTCCAGCATAGATTAAGTCTCCCATACGAAGGATTGATTAGCTCGAGACTTAATGTTATATACAAAATTCTTATACATGTTCTAGATCAGGATTGTTGCGCTACACTGTCTTCCATGTCGATCTTCTATATTCAGTTTTTGGCACAGTGATCGTCACTGTAATCTCAATAGATTTGAATTTCAATACTTCTATGGGAGGAGGATGAGTAGAAAGGTGTCCAAAGCGCTGGATCTATGGCTGCAACTCCTCATGCTTGTAAGCACCCGACAGCCAATTCGATCTACCTGACCTGATGCGGTATTCGAACTAAAAGCACAGATCAAGCACTGTTTCACACGATCTGAAGCACTGTTCTACTGCAAAGATAAGTGCATATTAGTTATTTTTCATACCGATCTTCACTGCGACGGTTTAGCGATCTTCATTATTTTCCACCTTCACCCCTCAACCCAGGGAGTGCGCTTTTCCCCGTCGAGATAAAATTACTAAATGTAAACTTTGCATGCAATTATGTAGCTGTATGGTAAGCAAATAAAAATCTTTCCTCCTATTGCAACACGTCGCTTGTTTGCAGCGGATATTTGCCCACAGTAAAGCCCGATCGCCTGTGCATTGTGTTTAACACGTGCTGCAAGCTGTATCAATACCGATCGATTGTATGATTCCACGATCTTCGTGTGGTATAAAAACGGCGCTGAACAGCAGTAAAGATCATCACTTACATTTGTTTTATTCTCCACGGAGTCAGTGAAATAATACCACAACAACAAAAAAACG

***trio* promoter sequence (AGAP001374, 1537 bp):**

TTTTCTGAGGTGATCTTTCGAAAAGATCGATCGACCGACCCCCAACGGCAACTCTCCCTCGGATGACCTCAGCCCAGAATAGGCATTCTTTCAGCAAGTCATTCTATCATCCCTTTTACATCTTACGTTACTCCAAAAAAAGAGAAGCTTTCCACCCCAAAGCTTTCGTTGTGGTGCAGCTTTCGATGGTGATCGCGATCACGGTACCACACGTACGTTTGTTTGCTACTCTCGCTCACCGTTTTCTTCGATTTCGTGTCTCGGGTACCGTTTGCTCGGTGCGGTAGTGCTGCGAAGAAAGCCGTTGGCCGAGAGAATAATGCTCACTTCGGCTGCACGCAACTCGCCGTGCCACAGCCGTGGAAGGGGTAGGTTGGATGGTCGGGGTCGGGACCGCAACGCCGAAGTGAACGCCGGCGTGCCAGCGAACGATCGCACCCCGGCACAGTCCAACTGCGTCGCTGGGCGCGCTCGGCGGACGGTTCTCGGTGTGTGCGCCCCCTTTGGTGCTCATCCCTCATATCTCATCTCATCCTTTCGCGCTGCTCGCGGTTCCCGCAGAACCACGCGCGAGAGGGAGTTGCGCGAACCAGGGAGAGCCCGACACCTGGGCAGCGTCCAGGCGGACCTGTTCGGGAACGGTCGCGGTGTATAAAACTCGATTCCCGCCCGCCGTTAGGTAACAGTCCATTGTGTAACGTTGCCGGGATCGCGAGCCATCCTGCAGGAACACAGCCGTGCGAGTTTGTGTGCGCGTGTATTTTGCCTACAAGCAAGTGAGCAGCAATCAGTGAGAACAACATCTAAAACAGAAAAACAAACAGTGAACTACAGTGCTGTGGAGCAGTGGAAATTGGTGTTAATTGAAGAGCTTTGTACATCGATCACACGGACAACCACGCACGTGGAGAAAGTTCTGCTGCGGTGCGGGTTACTTGCGTACACCACATTAAACATTGTGTGTGGCATTTAAAATAAATATACTCGCGCCCTCCCCTCAGCTGCTCTCGCGGGCACGCCCGATGCCAGAGGTCGCCCCGACCGGACATTGAACGTGGAAAGAAGAAAGTTGTCGGTTGTTTACACTTCCGTCCTGAGTGGTGTTGTGGCTACCGTTTGTGACCGTTGGTGAAATAAAGTAACATACCCCTCCCGAGGGCAGCAGCGACAATGACCTCCGGAAATTCGATAACAATACGTAAGTTACTTTCATTTTTTGCAAATTAAATTACCTCGTGATAATTGAGTGGAGTGAGTAGGTACAGGTTGTTAACGCGTCTTTTGTTTAGTTCGGCAATCCGGATCCGAAGATGAGAGGAGATTGCCTCTGATGATGATGTTATCATGATTGCTTGTGTAACGACGCAAAGATTCTCAGAGAACAACTTCTCCCAACATAATTAATCTTGTTGAAGGGTCTGTAAACACGTGGTGGGATATCTTACAACTGAACCAATATTCGACCGATAGTATAAAGTCCTTCGCACCATCGATGCGTTTACCATTTTCCCATAAAATCAATCTGCTCCCAGTTACA

***sag* 5' upstream sequence without promoter activity (AGAP000610, 220 bp):**

CTTTCATGCGTACAATGTGTATGATCACTGATGACAAAGCGCTTGGGTGGGCGCTTGCTGCTGAAGCGTGGCTGACAAATGGGGGGGGGGGGGGCTTGAACAAATATTTGCACACACGATAATGCTCTTGTTTGCCATGCGCTGACGGAAGACAAATCGCTGCAAGCGACGGCTCAAACCTGCTCAAACCAGGGAAGGTTTGCATAAGGTTCACTTATAA
