# Supplementary material for: A toolbox of engineered mosquito lines to study salivary gland biology and malaria transmission
Source: PLoS Pathog. 2022 Oct 12;18(10):e1010881. doi: 10.1371/journal.ppat.1010881 (PMC9555648; doi:10.1371/journal.ppat.1010881)
Supplement: Attachment — Submitted filename: 119784_1_rebuttal_2403436_rplcrl.pdf [file ppat.1010881.s013.pdf]

Full Revision

Manuscript number: RC-2021-01016
Corresponding author(s): Dennis Klug

1. General Statements [optional]

Dear editor, dear reviewers,

thank you very much for the quick review of our manuscript as well as for the constructive criticism and the interesting discussion of our results. Reading the comments, we realized that we may have put too much emphasis on the *in vivo* microscopy of sporozoites and their interaction with the salivary gland. We believe that the generated mosquito lines can be used to address different scientific questions, the *in vivo* microscopy of host-pathogen interactions being only one of them. Because of this imbalance, and to address some of the reviewers' comments, we have partially rewritten the manuscript (particularly the introduction). At the same time, we have implemented additional data on the inducibility of the promoters used, as well as on the functionality of hGrx1-roGFP2 in the salivary glands. Furthermore, we created an additional figure to better present the expression patterns of *trio* and *saglin* promoters within the median lobe, and we expanded the section on *in vivo* microscopy of sporozoites. We hope that these results further highlight the significance of our study. Accordingly, we have also changed the title of the manuscript to „A toolbox of engineered mosquito lines to study salivary gland biology and malaria transmission" to indicate the broad applicability of the generated mosquito lines and we have included an additional co-author, Raquel Mela-Lopez, who conducted the redox analysis. We hope that these changes will adequately answer the questions of the reviewers and address any concerns they may have had. We look forward to hearing from you.

With our kind regards,

Dennis Klug
Katharina Arnold
Raquel Mela-Lopez
Eric Marois
Stéphanie Blandin

2. Point-by-point description of the revisions

Reviewer #1 (Evidence, reproducibility and clarity (Required)):

Summary

This manuscript reports the generation and characterization of transgenic lines in the African malaria mosquito Anopheles coluzzii that express fluorescent proteins in the salivary glands, and their potential use for in vivo imaging of Plasmodium sporozoites. The authors tested three salivary gland-specific promoters from the genes encoding anopheline antiplatelet protein (AAPP), the triple functional domain protein (TRIO) and saglin (SAG), to drive expression of DsRed and roGFP2 fluorescent reporters. The authors also generated a SAG knockout line where SAG open reading frame was replaced by GFP. The reporter expression pattern revealed lobe-specific activity of the promoters within the salivary glands, restricted either to the distal lobes (aapp) or the middle lobe (trio and sag). One of the lines, expressing hGrx1-roGFP2 under control of aapp promoter, displayed abnormal morphology of the salivary glands, while other lines looked normal. The data show that expression of fluorescent reporters does not impair Plasmodium berghei development in the mosquito, with oocyst densities and salivary gland sporozoite numbers not different from wild type mosquitoes. Salivary gland reporter lines were crossed with a pigmentation deficient yellow(-) mosquito line to provide proof of concept of in vivo imaging of GFP-expressing P. berghei sporozoites in live infected mosquitoes.

Major comments

Overall the manuscript is very well written with a clear narrative. The data are very well presented. The generation of the transgenic mosquito lines is elegant and state-of-the art, and the new reporter lines are thoroughly characterized.

This is a nice piece of work that is suitable for publication, although the in vivo imaging of sporozoites is somewhat preliminary and would benefit from additional experiments to increase the study impact.

We would like to thank the reviewer for his/her appreciation of our manuscript. In the revised version, we have included additional experiments on *in vivo* imaging of sporozoites, which allowed us to quantify moving and non-moving sporozoites imaged under the cuticle of live mosquitoes. Although this is still a proof of concept, we believe that these new data provide novel interesting data and will better illustrate potential applications.

The reporter mosquito lines express fluorescent salivary gland lobes, yet the authors only provide imaging of parasites outside the glands. It would be relevant to provide images of the parasite inside the fluorescent glands.

We have now included images showing sporozoites inside the salivary glands *in vivo* in Figure 8C and discuss possible ways to further improve resolution and efficiency of the imaging procedure in lines 563-586.

The advantage of the pigmentation-deficient line over simple reporter lines is not clear, essentially due to the background GFP fluorescent in figure 5C. Imaging of GFP-expressing parasites should be performed in mosquitoes after excision of the GFP cassette under control of the 3xP3 promoter. This would probably allow to document the value of the reporter lines more convincingly.

Indeed, by incorporating two Lox sites in the transgenesis cassette, we designed the *yellow(-)KI* line to permit removal of the fluorescent cassette and completely exclude expression of the transgenesis reporter EGFP. Still,

EGFP expression in the *yellow(-)KI* adults is restricted to the eye and ovary, as we show now in Figure 7 supplement 1D. In contrast, no EGFP fluorescence was observed in the thorax area (Figure 7 supplement 1D). Therefore, we believe that the benefit of removing the fluorescence cassette for this study is limited. Moreover, the generation of such a line would take at least 3-4 months before experiments could be performed. Nevertheless, we agree with the reviewer that removal of the fluorescence cassette would be instrumental for follow-up studies. To draw the reader's attention to this issue, we now discuss background fluorescence in lines 378-387.

Along the same line, it is unclear if the DsRed spillover signal in the GFP channel is inherent to the high expression level or to a non-optimal microscope setting. This is a limitation for the use of the reporter lines to image GFP-expressing parasites.

We have discussed this problem with the head of the imaging platform at our institute, and we believe that it is not a problem that occurs due to incorrect settings. Rather, it seems to be due to the significant expression differences of the two fluorescence reporters used. We agree with the reviewer that this is a limitation and discuss the problem now in lines 416-412 and 565-567.

The authors should fully exploit the SAG(-) line, which is knockout for saglin and provides a unique opportunity to determine the role of this protein during invasion of the salivary glands. This would considerably augment the impact of the study. In this regard, line 131 and Fig S3E: why is there persistence of a PCR band for non-excised in the sag(-)EX DNA?

We definitely share the reviewer's enthusiasm about saglin and its role in parasite development in mosquitoes. We have thoroughly characterized the phenotype of *sag(-)* lines with respect to fitness and *Plasmodium* infection. These results are described in a spearate manuscript currently in peer review and available as a preprint on bioRxiv (https://doi.org/10.1101/2022.04.25.489337). Furthermore, in the revised manuscript, we have included additional data on the transcriptional activity of the *saglin* promoter with respect to the onset of expression and blood meal inducibility (Figure 2). In addition, we have included a completely new Figure 3 to highlight the spatial differences in transcriptional activity of the *saglin* promoter compared with the *trio* promoter. These new data are commented in lines 206-276.

There might be a misunderstanding in the interpretation of the genotyping PCR. The PCR shown in Figure 1 – figure supplement 3, displays PCR products for different genomic DNAs (*sag(-)EX*, *sag(-)KI* and wild type) using the same primer pair. „Excised" refers to *sag(-)EX* while „non excised" refers to *sag(-)KI* and „control" to wild type. Primers were chosen in a way to yield a PCR product as long as the transgene has integrated, only the shift in size between „excised" and „non excised" indicates the loss of the 3xP3-lox fragment. We have now changed the labeling of the respective gel in Figure 1 – figure supplement 3 to make this clearer.

Did the authors search for alternative integration of the construct to explain the trioDsRed variability?

We validated *trio-DsRed* cassette insertion in the *X1* locus by PCR. The only way to rule out an additional integration of the transgene would be whole genome sequencing, which we did not perform. Still, we believe that the observed expression patterns are due to locus-specific effects of the *X1* locus. Indeed, several lines of evidence point in this direction: (1) transgenesis was realized using the phage Φ31 integrase that promotes site-specific integration (*attP* is 38bp long and very unlikely to occur as such in the mosquito genome) and for which we never detected insertion in other sites in the genome for other constructs inserted in *X1* and other docking lines; (2) additional unlinked insertions would have been easily detected during the first backcrosses to WT mosquitoes we perform in order to isolate the transgenic line and homozygotise it; (3) we have often

observed variegated expression patterns for other transgenes located in the *X1* locus in the past, leading us to believe that this locus is subjected to variegation influencing the expression of the inserted promoters. Usually, the variation we observe is simpler (e.g. strong and weak expression of the fluorescent reporter placed under the control of the *3xP3* promoter in the same tissues where it is normally expressed), but some promoters are more sensitive to nearby genomic environment than others, which we believe is the case for *trio*. Finally, should there be additional insertions of the transgenesis cassette in the genome, they should all be linked to the *X1* locus as we would otherwise have detected them in the first crosses as mentioned above, which is unlikely. Thus, although very unlikely, we cannot exclude a single additional and linked insertion possibly explaining the high/low DsRed patterns, but variegation would still be required to explain other patterns. We have mentioned this alternative explanation in the manuscript in lines 522-524.

Line 254-255. Does the abnormal morphology of SG from aapp-hGrx1-roGFP2 result in reduced sporozoite transmission?

This is an interesting question. For future experiments, it could indeed be important to test if the transmission of sporozoites by the generated salivary gland reporter lines is not impaired. However, the quantification of the number of sporozoites in *aapp-hGrx1-roGFP2* expressing salivary glands did not reveal any significant differences from the wild type (Figure 5 – figure supplement 1B) and would definitely be sufficient to infect mice. As we have no evidence for reduced invasion of sporozoites in the salivary glands of *aapp-hGrx1-roGFP2* and of the *DsRed* reporter lines, no good reason to believe that the expression of fluorescent proteins would interfere with parasite transmission, and as we produced these lines as tools to follow sporozoite interaction with salivary glands, we have not performed transmission experiments.

Of note, we have now included images of highly infected salivary glands of all reporter lines in Figure 5 – figure supplement 1D to confirm that expression of the respective fluorescence reporter does not interfere with sporozoite invasion. Also we have not observed that sporozoites do not invade salivary gland areas displaying high levels of hGrx1-roGFP2.

Minor comments

-Line 51: sporogony rather than schizogony

Schizogony was replaced with sporogony.

-Line 56: sporozoites are not really deformable as they keep their shape during motility

This sentence was removed.

-In the result section, it is not clearly explained where constructs were integrated.

We have now included the sentence „...with an attP site on chromosome 2L...“ (line 173) and the respective reference (PMID: 25869647) to give more information about the integration site.

Line 106 and 434-435: for the non-expert reader, it is not clear what X1 refers to, strain or locus for integration?

X1 refers to both, the locus and the docking line. We have rephrased the beginning of the result section (previously line 106) to give more information about the integration site as mentioned above.

-Line 112-115: the rational of integrating GFP instead of SAG is not clearly explained here, but become clearer in the discussion (line

We have slightly rephrased the sentence to better explain the reasoning for this procedure (lines 182-184).

-Line 140: FigS2A instead of S3A

This mistake was corrected in the revised manuscript.

-Perhaps mention that GFP reporters (SG) might be useful to image RFP-expressing parasites.

We have now included an image of the *aapp-hGrx1-roGFP2* line infected with a mCherry expressing *P. berghei* strain in Fig. 7D.

-Line 236: the authors cannot exclude integration of an additional copy (as mentioned in the discussion line 367-368).

As discussed above, we removed „..as a single copy..." and introduced the possibility of an additional integration linked to X1 (lines 522-524).

-Line 257-258. The title of this section should be modified as SG invasion was not captured.

The title was rephrased. It reads now „Salivary gland reporter lines as a tool to investigate sporozoite interactions with salivary glands" (line 356-357).

-Line 287: remove "considerable number" since there is no quantification.

This was removed. In addition, we included new data in this section of the manuscript and rephrased the results accordingly (lines 406-427).

-Line 400-402: Klug and Frischknecht have shown that motility precedes egress from oocysts (PMID 28115054), so the statement should be modified.

Thank you for this suggestion. The passage was modified accordingly.

-Line 404: remove "significant number" since there is no quantification.

This section was rephrased and the phrase "significant number" was removed (lines 406-427).

-Line 497: typo "transgenesis"

The typo was correct in the revised manuscript.

-FigS1: add sag-DsRed in the title

Thank you for spotting this inconsistency, we corrected this mistake (line 1134).

-Stats: Mann Whitney is adequate for analysis in fig 2C but not 2B, where ANOVA should be used (more than 2 groups).

We have performed now an one-way-ANOVA test and adapted figure and figure legend accordingly.

Reviewer #1 (Significance (Required)):
This work describes a technical advance that will mainly benefit researchers interested in vector-Plasmodium interactions. Invasion of salivary glands by Plasmodium sporozoites is an essential step for transmission of the malaria parasite, yet remains poorly understood as it is not easily accessible to experimentation. The development of transgenic mosquitoes expressing fluorescent salivary glands and with decreased pigmentation provides novel tools to allow for the first time in vivo imaging in live mosquitos of the interactions between sporozoites and salivary glands.

Reviewer's expertise: malaria, Plasmodium berghei, genetic manipulation, host-parasite interactions

Reviewer #2 (Evidence, reproducibility and clarity (Required)):

The first achievements of the Klug et al. study are the (i) genetical engineering of the Anopheles coluzzii mosquitoes reared in insectarium, that stably express distinct fluorescent reporters (DsRed and hGrx1-roGFP2 and EGFP) under the putative "promoters" of genes reported to encode proteins expressed differentially in the pluri-lobal salivary glands(Sg) of anthropophilic blood-feeding adult females, (ii) the analysis of the promoter activity - based on the selected fluorescent reporter - with a primary focus on the salivary gland/Sg (including at the Sg lobe level) of the adult female but also considering the preimaginal developmental time with larvae and pupa samples. Of note, some data confirm the already reported time-dependent and blood meal-dependent promoter activity for the related Anopheles species. The last part presents preliminary dataset on live imaging of Plasmodium berghei sporozoites with the aim of highlighting the usefulness of these A. coluzzii transgenic lines to better understand how the rodent Plasmodium sporozoites first colonize and then settle as packed cells in Sg acinar host cells.

Major comments

The two first objectives presented by the authors have been convincingly achieved with (i) the challenging production of four different lines expressing different single or double reporters chosen by the authors (and appropriately presented in the result text and figure sections), (ii) the careful analysis of the spatiotemporal expression of the DsRed reporter under two "promoters" studied and with regards to the blood feeding event parameter. However, if the reason why the authors have put so much effort in the production of their transgenic mosquitoes is (and as mentioned) to provide a significant improved setting enabling the behavioral analysis of sporozoites upon colonization and survival in the Sg, it seems this part is kind of limited. Likely in relation with this perception is the fact I found the introductory section often confusing and not enough direct to the points: in particular distinguishing the rationale from the necessity to produce appropriate models, and clarifying what is/are the added value(s) offered by these new transgenic lines models when compared to what exist (in Anopheles stephensi) with specific evidence that argue for this knowledge gain. At this stage, it is unfortunately not clear to me, what is the bonus of imaging the Plasmodium fluorescent sporozoites in hosts with fluorescent salivary gland lobes if one can not monitor key events of the Sg-sporozoite interaction that were not reachable without the fluorescent mosquito lines. Furthermore, it should be better explained why the rodent Plasmodium species has been chosen rather Plasmodium falciparum (or other human species) for which A. coluzzii is a natural host; may be just mentioning that this study would serve as a proof of concept but bringing real biological insights would be fine.

We would like to thank the reviewer for his/her evaluation of our manuscript, which has helped us clarify our manuscript on several points. Our goal here was a proof of concept demonstrating potential applications for the fluorescent salivary gland reporter lines and for the low pigmented *yellow(-)* line we generated. *In vivo* imaging of sporozoites in salivary glands is one possible application that we intended to use as proof-of-concept, but we tailored the manuscript too restrictively with this aim in mind and neglected other applications as well as characterization of the biology of salivary glands in general. To improve this, we have included further data on the blood inducibility of the promoters tested (Figure 2), the functionality of roGFP2 in the salivary glands (Figure 5), and the use of the generated lines in the examination and definition of expression patterns of salivary gland proteins *in vivo* (Figure 6). Accordingly, we have adjusted the entire manuscript to adequately describe all the results presented. We have also rephrased major parts of the abstract and the introduction to better describe the impact of salivary gland biology on the transmission of pathogens, and to explain the anatomy of salivary glands in more detail.

We agree with the reviewer that it would be desirable to show direct salivary gland-sporozoite interactions *in vivo.* Still we believe that having mosquito lines expressing a fluorescent marker in the salivary gland as well as weakly pigmented mosquitoes are a first step to make this visualization possible, although we cannot provide a lot of quantitative data about this interaction yet.

1- The three genes and gene products selected by the authors should definitively be more systematically explained, which means for example the authors need to introduce the different mosquito species and the parasite-mosquito host pairs they are then referring to for the promoter/encoded proteins of their interest. In the same vein, I did not find any information as to the choice of the mosquito species (A. Coluzzii) for the current work. I was curious to know what is the advantage since better knowledge was available with Anopheles stephensi with respect to (i) Saglin and its promotor activity, (ii) aap driven dsRed expression (lines already existing) and (iii) sporozoite-gland interaction.

We have largely reworded the introduction to clarify the rationale for selecting these three promoters while providing a better understanding of salivary gland biology in general.

The choice of the mosquito species depends, in our opinion, strongly on the perspective and on the experiments to be performed. We agree with the reviewer that the malaria mosquito *A. stephensi* is a widely used model, based on its robustness in breeding and its high susceptibility to *P. berghei* and *P. falciparum* infections. However, in these cases, both vector-parasite pairs are to some extend artificial. Indeed, although it is also a vector of *P. falciparum* in some regions, *A. stephensi* mostly transmits *P. vivax* that cannot be cultured *in vitro*. Thus research efforts on this vector-parasite pair is limited. Also, due to the emerging number of observed differences between *Anopheles* species and their susceptibility to *Plasmodium* infection and transmission, more research has recently been conducted on African mosquito species. This effect is also reinforced by the fact that *P. falciparum*, unlike all other *Plasmodium* species infecting humans, causes the most deaths, making control strategies for species from the *A. gambiae* complex such as *A. coluzzii* particularly important. As a result, the number of available genetic tools in *A. coluzzi/gambiae* has overpaced *A. stephensi*. These include mosquito lines with germline-specific expression of Cas9 for site-directed transgenesis, lines expressing Cre for lox-mediated recombination, and several docking lines. Such tools are, as far as we know, not available in *A. stephensi* and were essential in reaching our objectives. Docking lines are of particular interest because they allow reliable integration into a characterized locus, which is an advantage over random transposon-mediated integration. Random insertion sites have generally not been characterized in the past, which can cause problems since integrations regularly occur in coding sequences. Docking lines also enable comparison of different transgenes as they are all integrated in the same genetic environment, which does not ensure some expression variation as illustrated in our manuscript. For all these reasons, we have thus chosen to work with *A. coluzzii*.

Concerning the use of the murine malaria parasite *P. berghei* instead of the human one *P. falciparum*, there are two reasons that motivated our choice. (1) For *in vivo* imaging of sporozoites, we needed a parasite line that is strongly fluorescent at this stage, and there is no such line existing for *P. falciparum*. Actually, there is no fluorescent *P. falciparum* line able to efficiently infect *A. coluzzii* reported thus far, as reporter genes have all been inserted in the *Pfs47* locus that is required by *P. falciparum* for *A. coluzzii* colonization. (2) Imaging *P. falciparum* infected mosquitoes, especially with sporozoites in their salivary glands, requires to have access to a confocal microscope in a biosafety level 3 laboratory. Hence our objective here was indeed to provide a proof of principle of *in vivo* imaging of sporozoites in the vicinity or inside salivary glands using our engineered mosquitoes, and to provide a first analysis of this process using *P. berghei* as a model of infection. Nevertheless, we agree with the reviewer that the goal should be to work as close as possible to the human pathogen.

Despite the wide range of topics that this study touches on, we want to try and keep the manuscript as concise as possible. Therefore, we have not discussed the advantages and disadvantages of the different vector-parasite pairs and ask the reviewer to indulge us in this.

2- To help clarifying the added value of the present study, introducing the species names of the mosquito and the Plasmodium that serve as a model would be appreciated.

We have included now the name of the used *Plasmodium* species in line 361. At this position we also give now more details about the transgene this line is carrying. We mention the used mosquito species *A. coluzzii* now at different positions in the manuscript (e.g. lines 52, 162 and 177).

3- Since a focus is the salivary gland of the blood feeding female Anopheles sp., a rapid description of the glands with different lobes and subdomains the results and figure 1 nicely refer to, would help in the introduction.

We explain now the anatomy of female and male mosquito salivary glands in the introduction (lines 119-123). The different lobes are now also indicated in the salivary gland images shown in several figures including Figure 1.

4- That description could logically introduce the few proteins actually identified with lobe specific or cell domain specific expression (apical versus basal side, intracellular or surface expose, vacuole, duct...) profiles. The context with regards to sporozoite biology would then easily validate the "promoter choice". As a minor remark, I miss the reason why the authors wrote " the astonishing degree of order of the structures (referring to the packing of sporozoites within the Sg acinars) raise the question whether sporozoite can recognize each other". Please clarify since packing/accumulation can be passive due to cell mechanical constraints and explain what this point has to see with the question and experimental work proposed here?)

We thank you for this suggestion. We have reworded key parts of the introduction to make the reasons for using the three selected promoters clearer. We also mention now other proteins expressed in the salivary glands which have been characterized in more detail because of their effect on blood homeostasis (e.g. anticoagulants) (lines 136-139).

The mention of stack formation of salivary gland sporozoites served only to clarify that almost nothing is known about the behavior of sporozoites within the salivary glands *in vivo* to explain why new methods are needed to make these processes visible. We have now reworded this passage to make this clearer, and we also mention that stack formation could also occur due to mechanical constraints, as suggested by the reviewer (lines 101-102, 106-110).

5- The selection of hGrx1-roGFP2 is quite interesting and justified but there is then no use of this reporter property in the preliminary characterization of the Sg and Sg-sporozoite interaction. Could the authors provide such characterization?

We have now implemented data testing the functionality of hGrx1-roGFP2 in the salivary glands. We also show qualitatively that the redox state of glutathione does not change upon infection with *P. berghei* sporozoites (Figure 6). We now describe and discuss these new data in lines 337-354.

6- Figure 1: it would be nice to add in the legend at what time the dissection/imaging has been made (age, blood feeding timing?). I would also omit the double mutant trio-Dsred/aapDsred in the main figure (may be

supplemental) since the two single mutants Dsred separately together with the double mutant (with different fluorescence) already provide the information. I would suggest to regroup the phenotypic presentation of the transgenic line made in the KI mosquitoes (current figure 5) in the main figure 1.

We have now added the missing information about the age of dissected mosquitoes and their feeding status in the legend of Figure 1. We also thank the reviewer for the suggestion to replace one image displaying aapp and trio promoter activity in trans-heterozygous mosquitoes with an image of the pigment deficient mutant *yellow(-)KI*. Still, due to the changes made to the manuscript based on the reviewers comments in general, we have now implemented new data highlighting the functionality of the generated salivary gland reporter lines investigating the redox state of glutathione as well as the expression pattern of the *saglin* and *trio* promoters at the single cell level (see Figure 3 and 6). Therefore it would no longer seem logical to introduce the *yellow(-)KI* mutant in Figure 1 while further data on this mutant are provided in the last two figures of the manuscript and discussed later in the manuscript (Figure 7 and 8). In addition we believe that co-expression of different transgenes (carrying fluorescent reporters) in the median and the distal lobes could potentially be interesting for certain applications. We believe that readers who might actually be interested in combining both transgenes in a cross would like to see the outcome to better evaluate the usefulness before experiments are planned and performed. This is especially true because localization as well as expression strength may differ between different fluorescence reporters while using the same promoter (e.g. the hGrx1-roGFP2 construct appears less bright and more localized to the apex of the distal-lateral lobes than dsRed, while expression of both reporters is driven by the *aapp* promoter in *aapp-hGrx1-roGFP2* and *aapp-DsRed*, respectively).

7- Figure 2:

a) Is there anything known on the Sgs' size change overtime. It seems that between day 1 and 2 there is an increase of size and volume as much as I can evaluate the volume (Fig S4). Could that mean that there is increase in cell number in the lobes and therefore more cells expressing the transgene which would account for the signal intensity increase rather than more transcripts per cell?

Thank you for this interesting question. The changes in the morphology of the salivary glands in *Anopheles gambiae* following eclosion have been studied in detail by Wells et al., 2017 (PMID: 28377572) which we cite now in the introduction (line 122-123). According to this reference, cell counts of the salivary gland are not changing upon emergence of the adult mosquito. However, we agree with the reviewer that the glands appear smaller and differ in morphology directly after eclosion. We noted that glands of freshly emerged females are more „fragile“ during dissections and lack secretory cavities, as reported by Wells et al., 2017. We believe that the increase in size occurs through the formation and filling of the secretory cavities which has been reported to take place within the first 4 days after emergence (Wells et al., 2017). This observation is in accordance with our observations that the promoters of the saliva proteins AAPP and Saglin display only weak activity after hatching, or, in the case of TRIO are not yet active directly after emergence. The timing of the formation of the secretory cavities is also in agreement with our time course experiment (Figure 2) which shows a strong increase in fluorescence intensity in dissected glands within the first 4 days after emergence.

b) why choosing 24h after the blood meal to assess promoter activity in the Sgs? Do we have any information on how the blood meal impact on the Sgs'development. At this time anyway the sporozoites are far from being made. Yosshida and Watanabe 2006 mentioned at significant decrease of Sg proteins post-blood feeding. Could the authors detail their rationale based on what the questions they wish to address

Thank you for this question. Unfortunately, the data available in the literature on this topic are very sparse, so we could only refer to few previous publications. The decision to quantify the fluorescence signals as early as

24 hours after blood feeding was based on Yoshida et al, Insect Mol. Biol, 2006, PMID: **16907827**. The authors of this study generated the first salivary gland reporter line in *A. stephensi* by using the *aapp* promoter sequence to drive *DsRed* expression, and showed by qRT-PCR that *DsRed* transcripts increase 1-2 days after blood feeding compared to controls. Consistent with this observation and because we were concerned that putative changes in protein levels would only be visible for a short period of time, we began quantification one day after feeding. Since we observed significant changes in fluorescence intensity for the *aapp-DsRed* and *sag(-)KI* lines 24 hours after blood feeding, we retained the experimental setup and did not change it further. Nevertheless, we agree with the reviewer that different time points could help determine how long the effect lasts, and whether trio expression might also be regulated by blood feeding, but at a later time point. Still, our main objective here was to validate that the ectopic expression of *DsRed* driven by the *aapp* promoter in the *aapp-DsRed* line was indeed induced upon blood feeding as previously reported (PMID: **16907827)**. This experiment allowed us to confirm the inducibility of *aapp* in a different way and to show for the first time that *saglin,* but not *trio,* is induced one day after blood feeding. Our transgenic lines could be used for follow-up studies investigating the inducibility of salivary gland-specific promoters by different stimuli, or after infection with *Plasmodium* sporozoites. For example, for *trio*, transcription has been shown to increase after infection of the salivary gland by *Plasmodium* (PMID: 29649443).

8- Figure 3: The figure is quite informative in terms of subcellular localization. Concerning the section "Natural variation of DsRed expression in trio-DsRed mosquitoes", I think it could be shortened because because it is a bit out of the focus the study.

We agree with the reviewer that this part of the manuscript sticks a bit out and is not perfectly in line with the remaining results because it doesn't deal with the salivary gland. Still, we would like to emphasise that in this work, we particularly want to show possible applications of the generated mosquito lines to address unanswered questions in host-parasite interactions and salivary gland biology. As a result, this manuscript establishes potentially important tools. For this reason, we feel it is important to mention the natural variation in *DsRed* expression, as this natural variation can have a significant impact on crossing schemes (especially with lines inheriting other DsRed-marked transgenes) and experiments (e.g. visualizing DsRed expression by western blot in larval and pupal stages). Furthermore, it is important for the use of the line to show that the transgene is inserted only once, at the expected location, which we try to emphasize with figure 4 – figure supplement 1 and figure 4 – figure supplement 2.

We would also like to note that transgenesis in *Anopheles* is a relatively young field of research and altered expression patterns of ectopically used promoters have rarely been described so far, although this could have major implications e.g. in the case of gene drives. Therefore, we hope that the data shown will bring this previously neglected observation more into focus and highlight the importance of accurate characterization of generated transgenic mosquito lines.

9- In contrast the last section of live imaging of P. berghei sporozoites in the vicinity and within salivary gland should be expanded. The 2 sentences summarizing the data are quite frustrating "We also observed single sporozoites moving actively through tissues in a back and forth gliding manner (Fig. 6B, Movie 3) or making contact with the salivary gland although no invasion event could be monitored"

We have now implemented new data and extended Figure 8 showing the results of the *in vivo* imaging in a qualitative manner. We have rephrased the result and discussion section accordingly.

10- I am aware of the technical difficulties to perform live imaging of sporozoite on whole mosquitoes, even when the salivary gland lobe under observation is closely apposed to the cuticle but that seems to be the final

aim of the authors. I looked very carefully to the three movies and I am sorry but at this stage I could not make meaningful analysis out of them, and could not agree with the conclusions: for instances, the authors specify that sporozoites were undergoing back and forth movements (movie 3) but I do not see that and do not see the Sg contours in the available movies? The authors should also add bar and time scales to their movies. Having an in-depth description with regards to the sub-domain marked by a relevant reporter would strengthen the study, even if images are not collected in the whole mosquito to get higher resolution.

We thank the reviewer for this comment. We have to admit that parasite imaging in fluorescent salivary glands *in vivo* is an ambitious goal given the complex biological system we are working with. We believe that the system presented in our manuscript is a first and important step to enable the analysis of the interaction of sporozoites with salivary glands, although in-depth analysis will require further optimization and considerable time, especially to generate quantitative data. Therefore, we now downstate the significance of our results in this respect and changed the title accordingly. Still, we also provide a more detailed analysis of the data we have already collected (Figure 8 and lines 406-427). Because we focus on the analysis of sporozoites in the thorax area in the revised manuscript, the outlines of the salivary gland are not necessarily visible in the images.

I am not sure I understand the relevance of this quite condensed sentence in the text. Could the authors rephrase and expand if they wish to keep the issues they refer to. "The sporozoites' distinctive cell polarization and crescent shape, in combination with high motility, allows them to „drill" through tissues". I would stress more on the main unknown in terms of sporozoite-Sg interactions and the need to get right models for applying informative approaches (i.e. here, imaging).

We thank you for this suggestion. The sentence mentioned has been removed in its entirety. We have also adjusted the text accordingly and reworded most of the introduction to make the narrative clearer (lines 91-119).

Of note, it could help to point that the "Sgs is a niche in which the sporozoites which egress from the oocyst could mature and be fully competent when co-deposited with the saliva into the dermis of their intermediary hosts"

We have now implemented a similar sentence in the introduction (lines 93-98).

Reviewer #2 (Significance (Required)):

1- Clear technical significance with the challenging molecular genetics achieved in the mosquito A. coluzzii.

2- More limited biological significance: fair analysis and gain of knowledge of spatio-temporal of reporter expression under the selected promoter but limited significance of the final goal analysis which concerns the Plasmodium sporozoite biology once egressed from oocysts

As stated above, we changed the title to place the focus on the engineered mosquito lines.

3- Previous reports cited by the authors have used the DsRed reporter and the aap promoter in another Anopheles (i.e. A. stephensi, Yoshida and Watanabe, Insect Mol Biol, 2006; Wells and Andrew, 2019) which is also a natural host and vector for human Plasmodium spp.) with significantly more resolutive 3D visualization of GFP-fluorescent P. berghei but in dissected salivary glands and not in whole mosquitoes. The Wells and Andrew publication entitled "Salivary gland cellular architecture in the Asian malaria vector mosquito Anopheles stephensi" in Parasite Vectors, 2015 would deserve to be reference and described.

Thank you very much for this suggestion. We considered citing Wells and Andrews (PMID: 26627194). However, this reference focuses very specifically on the subcellular localization of AAPP and shows only highly magnified sections of immunostained dissected and fixed salivary glands. Working only with the AAPP promoter, we felt it important to refer to the previously observed expression pattern along the entire salivary gland, as shown in Yoshida and Watanabe (PMID: 16907827). Nevertheless, we have cited two other publications by Wells and Andrews (PMID: 31387905 and 28377572) at various points in the manuscript.

4- Audience: I would say that this work should be of interest of mostly scientists investigating Plasmodium biology (basic and field research) or in entomology of Diptera.

5- To describe my fields of expertise, I can refer to my extensive initial training in entomology including at one point in the genetic basis of mosquito-virus interaction. I have also been working for more than 20 years in the field of Apicomplexa biology (Plasmodium and Toxoplasma) and I have long-standing interest in live and static high-resolution imaging.

Reviewer #3 (Evidence, reproducibility and clarity (Required)):

Klug et al. generated salivary gland reporter lines in the African malaria mosquito Anopheles coluzzii using salivary gland-specific promoters of three genes. Lobe-specific reporter activity from these promoters was observed within the salivary glands, restricted either to the distal lobes or the medial lobe. They characterized localization, expression strength and onset of expression in four mosquito lines. They also investigated the possibility of influences of the expressed fluorescent reporters on infection with Plasmodium berghei and salivary gland morphology. Using crosses with a pigmentation deficient mosquito line, they demonstrated that their salivary gland reporter lines represent a valuable tool to study the process of salivary gland colonization by Plasmodium parasites in live mosquitoes. SG positioning close to the cuticle in 20% of females in this strain is another key finding of this study.

The key findings from this study are largely quite convincing. The authors have created a suite of SG reporter strains using modern genetic techniques that aid in vivo imaging of Plasmodium sporozoites.

Vesicular staining within salivary acinar cells should be stated as "vesicle-like" staining unless a co-stain experiment in fixed SGs is conducted using antisera against the marker protein(s) and antisera against a known vesicular marker (e.g. Rab11). It may also be possible to achieve this in vivo using perfusion of a lipid dye (e.g. Nile Red), but this is not necessary. As is, in Fig. 3A, there are images in which it appears that the vesicle-like staining is located both within acinar cells' cytoplasm and in the secretory cavities (e.g. Fig. 3A: aapp-DsRed bottom and middle), and this is fine, but should be more inclusively stated. Fixed staining of the reporter strain SGs would allow for clarification of this point. In previous work, other groups have observed vesicle-like structures in both locations (e.g. PMID: 33305876).

Thank you very much for this suggestion. Indeed, when we observed the vesicle-like localization, we had similar ideas and considered investigating the identity of the observed particles in more detail. Ultimately, however, we concluded that the localization of DsRed does not play a critical role in the use of the lines as such and believe that a more detailed investigation of the trafficking of the fluorescent protein DsRed is beyond the scope of this study.

We have thus followed the suggestion of the reviewer and now use the phrase „vesicle-like" throughout the manuscript. In addition, we extended the discussion on the different localizations observed and presented some explanations that might have led to this observation. We also included a new reference that investigated the localization of AAPP using immunofluorescence (PMID: 28377572).

Morphological variation is extensive among individual mosquito SGs, thought to impact infectivity, and well documented in the literature. The manuscript should be edited to make it much clearer (e.g. n = ?) exactly how many SGs, especially in microscopy experiments, were imaged before a "representative" image was selected from each data point and in any additional experiment types where this information is not already presented. Figure S8 is an example where this was done well. Figure 3A-B is an example where this was not well done. All substantial variation (e.g. "we detected a strangulation..." - line 189) across individual SGs within a data point should be noted in the Results. Because of the genetics and labor involved, acceptable sample sizes for minor conclusions may be small (5-10), but should be larger for major conclusions when possible.

Thank you for this comment. We have improved this point by specifying precisely the number of samples and of repetitions in the respective figure legends. For example, we have now quantified the proportion of moving sporozoites and report both the number of sporozoites evaluated and the number of microscopy sessions required (see Figure 8).

Thank you for this comment. We have improved this point by specifying precisely the number of samples and of repetitions in the respective figure legends. For example, we have now quantified the proportion of moving sporozoites and report both the number of sporozoites evaluated and the number of microscopy sessions required (see Figure 8). Regarding Figure 3, fluorescence expression and localization in salivary gland reporter lines was actually very uniform in each line. We added the following sentence in the legend of revised figures 3 and 5: "Between 54 and 71 images were acquired for each line in ≥3 independent preparation and imaging sessions. Representative images presented here were all acquired in the same session".

Sporozoite number within SGs has been shown to be quite variable across the infection timeline, by mosquito species, by parasite strain, in the wild vs. in the lab, and according to additional study conditions. The authors mention that the levels they observed are consistent with their prior studies and experience, but they did not utilize the reporter strains and in vivo imaging to support these conclusions, instead relying on dissected glands and a cell counter. It is important for these researchers to attempt to leverage their in vivo imaging of SG sporozoites for direct quantification, likely using the "Analyze Particles" function in Fiji. The added time investment for this additional analysis would be around two weeks for one person experienced in the use of the imaging software.

Thank you for this interesting suggestion. Indeed, it would be beneficial to use an imaging based approach to quantify the sporozoite load inside the salivary glands. We already used „watershed segmentation" in combination with the „Analyze Particles" function in Fiji on images of infected midguts to determine oocyst numbers. Still, we believe this analysis cannot be applied to images of infected salivary glands mainly because of differences in shape and location of the oocyst and sporozoite stages. Sporozoites inside salivary glands form dense, often multi-layered stacks. Because of this close proximity, watershedding cannot resolve them as single particles which could subsequently be counted. This creates an unnecessary error by counting accumulations of sporozoites as one, likely leading to an underestimation of actual parasite numbers. Furthermore, given that the proximity issue could be resolved e.g. by performing infections yielding lower sporozoite densities, another problem would be that infected salivary glands prepared for imaging are often slightly damaged leading to a leak of sporozoites from the gland into the surrounding. These leaked sporozoites are likely not included on images which would then be used for analysis, potentially leading again to an underestimation of counts. Since these issues are circumvented by the use of a cell counter, we believe that this method is still the method of choice in acquiring sporozoite numbers.

Nevertheless, we can understand the reviewer's concern that counts performed with a hemocytometer do not reflect the variability in the sporozoite load of individual mosquitoes. To highlight that all generated reporter lines can have high sporozoite counts, we have now included images of highly infected salivary glands for each line in Figure 7D.

This manuscript is presented thoughtfully and such that the data and methods could likely be well-replicated, if desired, by other researchers with similar expertise.

The statistical analysis is appropriate for the experiments conducted. It is currently unclear if some experiments were adequately replicated. That information should be added to the paper throughout where it is missing.

We do appreciate your comments on our efforts to give all required information for other laboratories to replicate our experiments. We have added the missing information about the number of independent experiments in the respective figure legends wherever appropriate.

Studies from multiple groups should be more thoroughly referenced when the authors are describing the "vesicle-like" staining patterns observed in SGs from reporter strains (e.g. Fig. 3A). Is this similar to the SG vesicle-like structures observed previously (e.g. PMIDs: 28377572, 33305876, and others)?

Thank you for this comment. We did not discuss this observation in detail in the first version of our manuscript because the observed localization was rather unexpected, as DsRed was not fused to the AAPP leader/signal peptide. The observed localization is therefore difficult to explain, however, we have expanded the discussion on this (lines 465-482) and now cite one of the proposed references (PMID: 28377572, lines 468-469).

There are minor grammar issues in the manuscript text (e.g. "Up to date" should be "To date"). The figures are primarily presented very clearly and accurately. One minor suggestion: In cases such as Fig. S2A images 3 and 6, where some of the staining labels are very difficult to read, please move all labels for the figure to boxes located directly above the image.

We are sorry for the grammatical errors we have missed in the first version of our manuscript. We have now performed a grammar check over the whole manuscript. We have also increased the font size of the captions in the above figures and tried to make them better readable by moving the captions over the images.

The data and conclusions are presented well.

Reviewer #3 (Significance (Required)):

This report represents a significant technical advance (improved in vivo reporter strain and sporozoite imaging), and a minor conceptual advance (active sporozoite active motility), for the field.

This work builds off of previous SG live imaging studies involving Plasmodium-infected mosquitoes (e.g. Sinnis lab, Frischneckt lab, etc.), addressing one of the major challenges from these studies (reliable in vivo imaging inside mosquito SGs).

This work will appeal to a relatively small audience of vector biology researchers with an interest in SGs. Many in the field still see the SGs as intractable, instead choosing to focus on the midgut due to ease of manipulation. Perhaps work like this will spark new interest in tangential research areas.

I have sufficient expertise to evaluate the entirety of this manuscript. Some descriptors of my perspective include: bioinformatics, SG molecular biology, mosquito salivary glands, microscopy, RNA interference, SG infection, and SG cell biology.

Reviewer #4 (Evidence, reproducibility and clarity (Required)):

Klug et al generated transgenic mosquito lines expressing fluorescent reporters regulated by salivary gland specific promoters and characterized fluorescent reporter expression level over the time, subcellular localization of fluorescent reporters, and impact on P. berghei oocyst and salivary gland sporozoite generation. In addition, by crossing one of the lines (aapp-DsRed) with yellow(-) KI mosquitoes, they open up the possibility to perform in vivo visualization of salivary glands and sporozoites.

Overall the generation and characterization of these transgenic lines is well-done and will be helpful to the field. However, there are several concerns with the in vivo imaging data shown in Figure 6, which does not convincingly show fluorescent sporozoites in the lobe or secretory cavity of a fluorescent salivary gland lobe. This needs to be addressed. Points related to this concern are outlined below:

(1) Although the authors mention that the DsRed signal was strong enough to see with GFP channel, it would be more appropriate to show that the DsRed signal from salivary glands and GFP channel image co-localize.

We now show a merge of the GFP and DsRed signal in Figure 7 – figure supplement 2 The yellow appearance of the salivary gland in the merge likely indicates the spillover of the DsRed signal into the GFP channel. In addition we discuss the issue in lines 416-412 and 565-567.

(2) Mosquitoes were pre-sorted using the GFP fluorescence of the sporozoites on day 17-21. From figure 4B, median salivary gland sporozoite number was about 10,000 sporozoites/mosquito on day 17-18. However, in Figure 6A there are no sporozoites in the secretory cavities. They should be able to see sporozoites in the cavities at this time. Can the authors confirm that they can visualize sporozoites in secretory cavities in vivo and perhaps show a picture of this.

This is entirely correct. We also examined mosquitoes for the presence of sporozoites in the salivary glands and wing joints prior to imaging, as shown in Figure 7B and Figure 7 – figure supplement 2A, to increase the probability that sporozoites could be observed. Nevertheless, the area of the salivary gland that comes to the surface is often small and limited to a few cells that can be imaged with good resolution. Unfortunately, these same cells were often not infected although other regions of the salivary glands must have been very well infected based on the previously observed GFP screening (Figure 7B). In addition, with the confocal microscope available to us, we struggled to achieve the necessary depth to image sporozoites in the cavities of the salivary gland cells. For this reason, we were often able to detect a strong GFP signal in the background, but not always to resolve the sporozoites sufficiently well. Still, we have now included an image showing sporozoites in salivary glands (Figure 8C). However, we believe that the method can be further improved to be more efficient and provide better resolution. We discuss possible ways to further improve the imaging in lines 563-586.

(3) There is no mention of the number of experiments performed (reproducibility) and no quantification of the imaging data. In the results (line 287-288), the authors state that sporozoites are present in tissue close to the gland and sometimes perform active movement. How can this be? Do they believe these sporozoites are on route to entering? More relevant to this study would be a demonstration that they can see sporozoites in the secretory cavities of the salivary gland epithelial cells, this should shown. If they have already performed a number of experiments, I would suggest to do quantification of the number of sporozoites observed in defined regions . The mention that sporozoites are moving is confounded by the flow of hemolymph. How do they know that the sporozoites are motile versus being carried by the hemolymph. Perhaps it's premature to jump to sporozoite motility in the mosquito when they haven't even shown sporozoite presence in the salivary glands.

Thank you very much for this comment. We have followed the suggestions of the reviewer and have now quantified the behavior of sporozoites in the thorax area of the mosquito. For the analysis, we only considered sporozoites that could be observed for at least 5 minutes. This analysis revealed that 26% of persistent sporozoites performed active movements, which in most cases resembled patch gliding previously described *in vitro*. We adjusted the results section accordingly. In addition, we have changed the figure legend to accurately indicate the number of experiments performed. Likewise, we now also provide an image of sporozoites that we assume are located in the salivary gland (Figure 8C). Although we have not yet been able to image and quantify vector-sporozoite interactions extensively (further improvements would be required, as mentioned previously), we believe these results illustrate the potential of the transgenic lines.

(4) In vivo imaging has been performed with the mosquito' sideways. Was this the best orientation? Have you tried other orientations like from the front (Figure 5B orientation).

It is true that in the abdominal view as shown in Figure 7B the fluorescence in the salivary glands is very well visible. This is mainly due to the fact that in this area the cuticle is almost transparent and therefore serves as a kind of "window". Nevertheless, the salivary glands are not close to the cuticle in this position, which makes good confocal imaging impossible. Imaging always worked best where the salivary gland was very close to the cuticle, and this was always laterally. However, there were differences in the position of the salivary glands in individual mosquitoes, which also led to slight differences in the imaging angle.

Overall, the text is easy to follow and I have only few suggestions.

Thank you for this comment.

In the result section, the authors describe the DsRed expression during development of mosquito (line 194-236) after they describe subcellular localization of fluorescent reporters. I felt the flow was disrupted. Thus, this part (line 194-236) could summarize and move to line 135. In this way, the result section flow according to the main figures.

Thank you very much for this suggestion. We have considered your idea, but based on the changes we have made in response to reviewer comments and new data implemented in the form of two new figures, we believe the current order in the results section is more appropriate. The rationale was primarily to first characterize the expression of fluorescent reporters in the salivary glands of all lines before going into more detail on expression in other tissues of a single line. We then finish with potential applications like *in vivo* imaging of sporozoite interactions with salivary glands.
Also, and as mentioned previously (reviewer 2, point 8), we believe it is important to describe the variability of ectopic promoter expression at a given locus with sufficient details, as this has not been characterized thus far despite its importance.

In the result section, text line 186-190, the authors describe the morphological alternation of salivary gland in aapp-hGrx1-roGFP2. I would suggest to mention that this observation was only in one of lateral lobe. (I saw that it was mentioned in the figure legend but not in the main text.)

We believe there has been a misunderstanding. The morphological alteration in salivary glands expressing *aapp-hGrx1-roGFP2* was observed in all distal-lateral lobes to varying degrees (quantification in Figure 6E). To include as many salivary glands as possible in the quantification and because in some images only one

distal-lateral lobe was in focus, only the diameter of one lobe per salivary gland was measured and evaluated. We have now revised the legend to prevent further misunderstandings.

In the discussion section, author discuss localization of fluorescent reporters (line 322-331). When I looked at aapp-DsRed localization pattern (Figure 3A), the pattern looked similar to the previous publication by Wells et al 2017 (https://www.nature.com/articles/s41598-017-00672-0). This publication used AAPP antibody and stain together with other markers (Figure 4-7). This publication could be worth referring in the discussion section.

Thank you for this suggestion. According to the information available through Vectorbase, we did not fuse DsRed with any coding sequence of AAPP that could potentially encode a trafficking signal. Therefore, it is rather unlikely that the observed DsRed localization in our aapp-DsRed line and the localization observed by AAPP immunofluorescence staining in WT mosquitoes match. This is further exemplified by the cytoplasmic localization of hGrx1-roGFP2 in the *aapp-hGrx1-roGFP2* line, where the reporter gene was cloned under the control of the same promoter. For this reason, we had not mentioned this reference in the first version of the manuscript. In the revised manuscript, we have included now the suggested reference (lines: 475-476) and extended the discussion on possible reasons which led to the observed localization pattern.

In the text, authors describe salivary gland lobes as distal lobes and middle lobe. It would be more accurate to refer to the lobes as the lateral and medial lobes. The lateral lobes can then be sub-divided into proximal and distal portions. I would suggest to use distal lateral lobes, proximal lateral lobes and median lobe as other references use (Wells M.B and Andrew D.J, 2019).

Thank you for this suggestion. We have corrected the nomenclature for the description of the salivary gland anatomy as suggested throughout the manuscript.

Overall, the figures are easy to understand and I have following suggestions and questions.
Figure 1C) It is hard to see WT salivary gland median lobe. If authors have better image, please replace it so that it would be easier to compare WT and transgenic lines.

We have replaced the wild-type images of salivary glands in this figure and labeled the median and distal-lateral lobes accordingly (see Figure 1).

Figure 2) While it was interesting to observe the significant expression differences between day 3 and day 4, have you checked if this expression maintained over time or declines or increases (especially on day 17-21 when author perform in vivo imaging)?

Thank you for this interesting question. We have not quantified fluorescence intensities in mosquitoes of higher age. Nevertheless, we regularly observed spillover of DsRed signaling to the GFP channel during sporozoite imaging, suggesting that expression levels, at least in *aapp-DsRed* expressing mosquitoes, remain high even in mosquitoes >20 days of age (see Figure 8A). We also confirmed this observation by dissecting salivary glands from old mosquitoes, whose distal lateral lobes always showed a strong pink coloration even in normal transmission light (data not shown).

Figure 3A) There is no description of "Nuc" in figure legend. If "nuc" refers to nucleus, have you stained with nucleus staining dye (example, DAPI)?

Thank you for spotting this missing information in the legend. Initial images shown in this figure were not stained with a nuclear dye. To test whether the observed GFP expression pattern really colocalizes with DNA,

Review COMMONS

we performed further experiments in which salivary glands from both *aapp-hGrx1-roGFP2* and *sag(-)KI* mosquitoes were stained with Hoechst. We have now included these new data in Figure 3 - figure supplement 1. It appears that GFP is concentrated around the nuclei of the acinar cells, which makes the nuclei clearly visible even without DNA staining.

Figure 4B) The number of biological replicates in the figure and the legend do not match (In the figure, there are 3-5 data points and, in the legend, text says 3 biological replicates.)

Thank you for spotting this inconsistency. The number of biological replicates refers to the number of mosquito generations used for experiments. The difference is due to the fact that sometimes two experiments were performed with the same generation of mosquitoes using two different infected mice. We have clarified the legend accordingly to avoid misunderstandings.

Figure 4C) The number of data points from (B) is 5. However, in (C) only 4 data points are presented.

We have corrected this mistake. In the previous version, the results of two technical replicates were inadvertently plotted separately in (B) instead of the mean.

Figure 5) I would suggest to have thorax image of P. berghei infected mosquito to show both salivary glands and parasites.

Thank you for this suggestion. Images in Figure 7B (previously Figure 5) were replaced with an infected specimen to show salivary glands (DsRed) and sporozoites (GFP) together.

Reviewer #4 (Significance (Required)):

The transgenic lines that authors created have potential for in vivo imaging of salivary gland and sporozoite interactions. Since the aapp and trio lines have distinct fluorescence expression, they could help elucidate why sporozoites are more likely to invade distal lateral lobes compare to median lobe.

My areas of expertise are confocal microscope imaging, mosquito salivary gland and Plasmodium infection and sporozoite motility.
