# Supplementary material for: A toolbox of engineered mosquito lines to study salivary gland biology and malaria transmission
Source: PLoS Pathog. 2022 Oct 12;18(10):e1010881. doi: 10.1371/journal.ppat.1010881 (PMC9555648; doi:10.1371/journal.ppat.1010881)
Supplement: Attachment — Submitted filename: PPATHOGENS-D-22-01272_rebuttal_letter_050922.docx [file ppat.1010881.s014.docx]

Reviewer's Responses to Questions

Part I - Summary

Please use this section to discuss strengths/weaknesses of study, novelty/significance, general execution and scholarship.

Reviewer #1: Klug et al. provides a detailed account of the generation of fluorescent reporter lines in the African malaria mosquito Anopheles coluzzii using the salivary gland-specific promoters of the anopheline antiplatelet protein (AAPP), the triple functional domain protein (TRIO) and saglin (SAG) genes. Promoter activity was specifically observed in the distal-lateral lobes or in the median lobe of the salivary gland. Besides a comparison of the expression patterns of the selected promoters, the fluorescent probes allowed evaluation of the inducibility of these three promoters upon blood feeding and to measure intracellular redox changes. They also combined the aapp-DsRed fluorescent reporter line with a pigmentation-deficient yellow(-) mosquito mutant in order to assess the feasibility of in vivo microscopy of parasitized salivary glands. This combination allowed location of the salivary gland through the cuticle and imaging of individual sporozoites in vivo, which facilitates live imaging studies of salivary gland colonization by Plasmodium sporozoites.

Strengths: The manuscript is well written, and the conclusions presented are both appropriate in scope and well-justified by the data presented and described within. The edits to the title greatly improve the reader's expectations for the scope of this research, and the tools that have been generated by these authors are both very useful and serve as an indicator of the current state of this field (goals and future directions).

Weaknesses: The limitations of this study result primarily from the relative deficiencies of standard microscope approaches and the mosquito as a research organism. Great future efforts will be needed to fully utilize molecular genetics approaches to better understand the journey of the sporozoite through into, through, and out of, the mosquito salivary glands. Specifically regarding this study, not using human malaria parasites is a key weakness, but understandable at this time. The authors provided an appropriate justification for their choices of mosquito and parasite species.

Novelty/Significance: The authors applied the most appropriate, cutting edge technologies (e.g. CRISPR/Cas9 editing) in meaningful ways in order to address the aim of this study. They also utilized meaningful historical approaches (e.g. yellow mutants) in innovative ways. While not exclusively novel, per se, these authors have conducted a well-designed and supported research study of great significance to the vector biology community that provides further insights into mosquito SG-Plasmodium interactions and provides the impetus for further methodological improvements and deeper future analyses.

Execution: The data presented by these authors is complete and rigorous, meeting or exceeding expectations for this level of inquiry. The figures are informative and presented clearly, labeled well, and represent sufficient experimental replicates to validate their results.

Scholarship: The authors demonstrate strong command of the historical and most current scientific literature relevant to their study. Appropriate citations have been included and are representative of the studies most closely linked to the introduction and conclusions of this work. The authors did a masterful job of reworking the introduction to better frame the scope of this research.

Reviewer #2: This is a revised version of a manuscript that I have previously reviewed for Review Commons (as reviewer #1). This manuscript reports the generation and characterization of transgenic lines in the African malaria mosquito Anopheles coluzzii, expressing fluorescent proteins in the salivary glands, and their potential use for in vivo imaging of Plasmodium sporozoites.

As already indicated in my previous report, this is a nice piece of work describing a clear technical advance. The generation of the transgenic mosquito lines is elegant and state-of-the art, and the new reporter lines are thoroughly characterized. In their revised version, the authors addressed all my comments. In particular, they provide new imaging data supporting the proof-of-concept that sporozoites can be visualized in live mosquitoes. Future studies using these new transgenic mosquito lines may bring novel biological insights into salivary gland colonization by malaria parasites.

Reviewer #3: The manuscript describes generation of fluorescent reporter lines in A. coluzzii using salivary gland-specific promoters such as AAPP, TRIO and SAG. The characterization of these lines from larvae to mosquitoes are well described and in-depth description of the fluorescent reporter expression in salivary glands are informative. Fluorescence signal of aapp-DsRed line localized in the distal lateral lobe, and trio-DsRed, sag (-)KI and sag(-)EX signal localized in the median lobe. More specifically, in the median lobe, stronger signal of sag (-)KI was observed in the base and trio-DsRed signal was stronger in the apex. While trio-DsRed fluorescence intensity was not blood feeding dependent, aapp-DsRed and sag(-)EX fluorescence intensity increased upon blood feeding. In addition, the authors showed the evidence of a functional hGrx1-roGFP2 probe and it is interesting that P. berghei infection does not affect redox status of salivary glands. Finally, authors performed in vivo imaging and successfully visualized the salivary gland and sporozoites. In vivo imaging requires further development as authors discussed, and together with current development of imaging techniques (e.g. [https://doi.org/10.1101/2021.10.15.463778](https://doi.org/10.1101/2021.10.15.463778) in bioRxiv), these lines will contribute to study the sporozoite-salivary gland interaction in the future.

We would like to thank all the reviewers and editors who took the time to read and evaluate our manuscript. We are pleased to see that their evaluations are all very positive. Below is our detailed response to the reviewers’ comments.

Please note that the line numbers given below refer to the Word file of the manuscript with tracked changes.

---------------------------------------------

Part II – Major Issues: Key Experiments Required for Acceptance

Please use this section to detail the key new experiments or modifications of existing experiments that should be absolutely required to validate study conclusions.

Generally, there should be no more than 3 such required experiments or major modifications for a "Major Revision" recommendation. If more than 3 experiments are necessary to validate the study conclusions, then you are encouraged to recommend "Reject".

Reviewer #1: I did not identify any major issues with this revised manuscript.

Reviewer #2: I find it surprising that despite heavy sporozoite loads in the glands only a few sporozoites could be imaged (Fig 8 and Fig 7supplt2). Is it merely a technical issue due to the difficulties in imaging deep tissues (such as salivary glands) in live mosquitoes? The authors should discuss further how imaging could be improved, in terms of microscope type, objectives, etc... This could be instrumental for full exploitation of the new tools they have generated in this work.

Indeed, the main limitation is the imaging depth that restricts the area and sporozoites that can be imaged in our study. After their successful infection of salivary glands, most sporozoites form bundles within the cavities of the acinar cells. Imaging these bundles would require peering through the cuticle, hemolymph fluid, and cell bodies of the acinar cell(s), which, if using confocal microscopy, would be possible only in rare occasions when glands are located very close to the cuticle. Hence, using confocal microscopy, the sporozoites that we were able to image with sufficient quality were those located in close proximity to a subset of salivary gland lobes, themselves ideally positioned close to the cuticle.

To address reviewer #2’s suggestion, we have now slightly expanded the discussion to mention potential imaging improvements, notably through the use of two-photon microscopy, a method better suited for imaging deep tissues (lines 646-651). Accordingly, we now also cite the reference suggested by reviewer #3 ([https://doi.org/10.1101/2021.10.15.463778](https://doi.org/10.1101/2021.10.15.463778)). We also included the following sentence (lines 651-653): “Sample selection and preparation, like partial embedding of mosquitoes as well as the choice of the immersion medium, could also lead to significant improvements.”

Reviewer #3: No new experiments required.

---------------------------------------------

Part III – Minor Issues: Editorial and Data Presentation Modifications

Please use this section for editorial suggestions as well as relatively minor modifications of existing data that would enhance clarity.

Reviewer #1: The figures are largely very clear. I identified two areas for minor improvements.

In Fig. 7C - creating a second version of lower right panel with dashed outline around the presumptive SGs might make this figure easier to interpret than arrows for non-experts.

Thank you for this suggestion. We have now included enlargements in a third panel of images in Fig. 7C to better illustrate that it is possible to distinguish oocysts through the darker parts of the cuticle of *yellow(-)* mutants. We kept arrowheads pointing at some of them as circling them is less visible on the picture.

In Fig. 8A quantification, comma/period use is inconsistent within the graph (right side).

We now use periods everywhere.

Reviewer #2: -I feel it would be more logical to place lines 380-390 after lines 391-397.

Thank you very much for this suggestion. We have swapped the two passages accordingly.

-Line 384: typo “surprisingly”

Corrected

-In fig7B there seems to be an auto-fluorescent structure in the GFP channel, which does not overlap with the salivary gland DsRed signal. This is in contradiction with Fig7-supplt1D where no such fluorescence is observed in the throat region. Are these really Pb sporozoites? Perhaps the authors could select a more convincing image.

Thank you for this question. We assume that reviewer #2 refers to the strong GFP signal that is detected in both WT and *yellow(-)-aapp-DsRed* mosquitoes in the throat region. This signal is indeed due to GFP-expressing sporozoites that accumulate in the salivary glands, which explains why it is absent in non-infected individuals (**S8D Fig.**). Of note, at the level of salivary glands, on the ventral side, the cuticle forms a window that is less pigmented (see bright-field images in **Fig. 7B**), which likely explains why the weak green signal (in median lobes not expressing *DsRed* – see the dissected salivary gland of aapp-DsRed in **Fig 7D**) is mainly visible within this window, whereas the red fluorescence expressed in the lateral lobes of the salivary glands is much stronger and visible through the more pigmented parts of the cuticle. Of note, in both WT and *yellow(-)-aapp-DsRed* mosquitoes presented in **Fig 7B**, the salivary gland median lobes (2 in each mosquito) appear located in front of the window while the lateral lobes are on the sides.

In addition, two green dots are also visible on both sides of the lower thorax of both WT and *yellow(-)-aapp-DsRed* mosquitoes in Fig. 7B. These are sporozoites that are stuck in the wing joint in infected mosquitoes and thus, this signal is also not detected in naïve ones (**S8D Fig.**). As mentioned in the manuscript (lines 394-397), sporozoites are often visible at this site when mosquitoes are heavily infected and carry high sporozoite loads. We have now clarified the nature of the green dots in the legend of **Fig. 7B** and included references to this figure when we mention the detection of sporozoites at the wing junction (lines 397 & 469).

-Line 409-410: invasion of the salivary glands begins before day 17

This is indeed correct. We have modified the sentence accordingly. It reads now „...between day 17 and day 20 after infection, when the majority of sporozoites have invaded the salivary glands.“ (line 443).

Reviewer #3: Line 101

Previous description on sporozoites in the cavities of the acinar cells used “bundles” (PMID: 7866385 also in Wells and Andrew, 2019). I would suggest to change “stacks” to “bundles” and add the reference (PMID: 7866385).

We have followed the reviewer's advice and changed "stacks" to "bundles" and added the recommended reference Pimenta et al., 1994 (lines 103-108).

Line 271-278

The description of number of cells in median lobe and quantification of high-low signal of is informative. However, I did not find the detailed method. Please add following information to Materials and Methods section.

• how you acquired z-stacks (increment)

• description of how you quantify cells

• description of how you determine high-low promoter activity

We are sorry that we forgot to include this important information. We have now added the requested experiment description in Material & Methods (lines 846-856).

Figure 6C

It is an interesting observation that there is no change in redox state in the salivary glands upon infection with P. berghei. Please add the number of experiments performed in the figure legend.

Sorry again for that, we added the missing information in the figure legend (lines 1192-1193).

Line 460-462

Please clarify these line. Especially, “the protein (saglin) has been shown to affect colonization of the salivary glands by Plasmodium sporozoite (Ghosh and Jacobs-Lorena, 2009)”. I assume these lines refer to the TRAP-saglin interaction during sporozoite invasion of salivary glands. If so, please change the reference to (Gosh, A.K. et al, 2009).

Thank you for spotting this mistake. We use now the correct reference Ghosh, A.K. et al, 2009 (line 528).

In addition to the changes suggested by the reviewers, we also corrected some typos or language mistakes, and made the following changes:

- mosquito line names corrected in lines 282 and 290
- lines 530-531 corrected to refer only to saglin, and not both saglin and trio
- MR4 catalog reference added for the G3 mosquito line (Key Resources Table)
- all references to Key Resources Table corrected (previously appearing as Tables S1 and S2 in the Materials and methods section)
- typo in a plasmid name corrected (line 760)
- acknowledgements and authors’contributions paragraphs slightly modified
- two labels in Fig. 1 which had slipped during the previous editing of the figure, have been correctly repositioned.
- captions of supplemental data changed to be appropriate for PLoS Pathogens
